# Species-Specific Cuticular Hydrocarbon Stability within European *Myrmica* Ants

**DOI:** 10.1007/s10886-016-0784-x

**Published:** 2016-11-10

**Authors:** Rhian M. Guillem, Falko P. Drijfhout, Stephen J. Martin

**Affiliations:** 1 Department of Animal and Plant Sciences, University of Sheffield, S10 2TN, Sheffield, UK; 2 Department of Earth & Life Sciences, Gibraltar Botanic Gardens Campus, University of Gibraltar, Gibraltar, GX11 1AA Gibraltar; 3 Chemical Ecology Group, School of Physical and Geographical Sciences, Lennard-Jones Laboratory, Keele University, Keele, ST5 5BG UK; 4 School of Environment & Life Sciences, University of Salford, Manchester, M5 4WT UK

**Keywords:** Chemotaxonomy, Cuticular hydrocarbons, *Myrmica*, Chemical recognition

## Abstract

Recognition is a fundamental process on which all subsequent behaviors are based at every organizational level, from the gene up to the super-organism. At the whole organism level, visual recognition is the best understood. However, chemical communication is far more widespread than visual communication, but despite its importance is much less understood. Ants provide an excellent model system for chemical ecology studies as it is well established that compounds known as cuticular hydrocarbons (CHCs) are used as recognition cues in ants. Therefore, stable species-specific odors should exist, irrespective of geographic locality. We tested this hypothesis by comparing the CHC profiles of workers of twelve species of *Myrmica* ants from four countries across Europe, from Iberia to the Balkans and from the Mediterranean to Fennoscandia. CHCs remained qualitatively stable within each species, right down to the isomer level. Despite the morphological similarity that occurs within the genus *Myrmica*, their CHCs were highly diverse but remarkably species-specific and stable across wide geographical areas. This indicates a genetic mechanism under strong selection that produces these species-specific chemical profiles, despite each species encountering different environmental conditions across its range.

**Electronic supplementary material:**

The online version of this article (doi:10.1007/s10886-016-0784-x) contains supplementary material, which is available to authorized users.

## Introduction

As the species category is a fundamental unit in biology, the central goal of species delimitation is to develop robust and highly replicable measures for identification. This has focused mainly on morphology-based delimitation, utilizing visual characteristics found in study species. However, in insects, the primary recognition system between individuals is via chemical cues, so it is predicted that species-specific chemical recognition should be stable across a species’ entire geographical range due to strong selection pressures. It is well established that cuticular hydrocarbons (CHCs) function extensively in chemical communication within insects and serve as recognition signals (e.g., Blomquist and Bagnères 2010). Despite this, chemical recognition remains less understood than visual recognition.

It has long been known that CHCs are species-specific (Blomquist and Bagnères 2010; Martin et al. 2008a) and can thus be readily exploited for use in chemotaxonomy (Berville et al. 2013; Page et al. 2002; Takematsu and Yamaoka 1999). However, in order to effectively exploit chemistry as a taxonomic tool we need to ensure that a species’ CHC profile is stable throughout its geographical distribution. A review of the CHCs of 78 species of ant found that no two species had the same combination of compounds, despite almost 1000 CHCs described (Martin and Drijfhout 2009). Despite this, few comparative studies exploring the CHCs of species across their geographical ranges exist (Berville et al. 2013; Bonelli et al. 2015; Martin et al. 2008a; Steiner et al. 2002), and none specifically investigates intra-specific variability across many geographic localities. If, as predicted, species-specific CHC profiles are stable across their entire range, then this will have benefits in identifying cryptic or sibling species (Akino et al. 2002; Lucas et al. 2002; Steiner et al. 2002). Since chemotaxonomy can outperform other taxonomic tools (e.g., Seppä et al. 2011) its value in the taxonomic and ecological world could potentially be beneficial, especially as current taxonomic methods such as morphological characters and mitochondrial genes can sometimes fail in delineating species (Seppä et al. 2011). In cases such as these, a multi-disciplinary approach is best (Schlick-Steiner et al. 2010; Seifert et al. 2013).

The ant genus *Myrmica* makes an ideal model system for testing ecological and evolutionary hypotheses, as species are abundant throughout Europe and have been well studied biologically, taxonomically and phylogeographically (Jansen et al. 2010; Radchenko and Elmes 2010; Seifert 1988). In addition, earlier studies on ant chemistry often used species from this genus (Attygalle et al. 1983; Evershed et al. 1982; Morgan et al. 1979). Limited studies on *Myrmica* have shown CHC profiles to be species-specific, at least within a small geographical range for a limited number of species (Elmes et al. 2002; Guillem et al. 2012). The *Myrmica* species used in this study were chosen because they are common and abundant, have extensive and continuous temperate-boreal Palaearctic distributions, and have wide ecological tolerances, occurring in a diverse array of habitats and climates. Species boundaries have been thoroughly tested morphologically and genetically, and all are currently considered to be good species (Radchenko and Elmes 2010). Their chemical profiles are relatively simple compared to some other genera, making them good candidates for a large-scale CHC study.

This study investigated the stability of CHC profiles of 12 species of *Myrmica* across large geographic distances (countries) and a wide range of habitats, with the aim of testing if stable species-specific CHC profiles exist irrespective of a variable environment, which is believed to play a major role in determining the production of CHCs.

## Methods and Materials

### Source Material

**Fig. 1:**
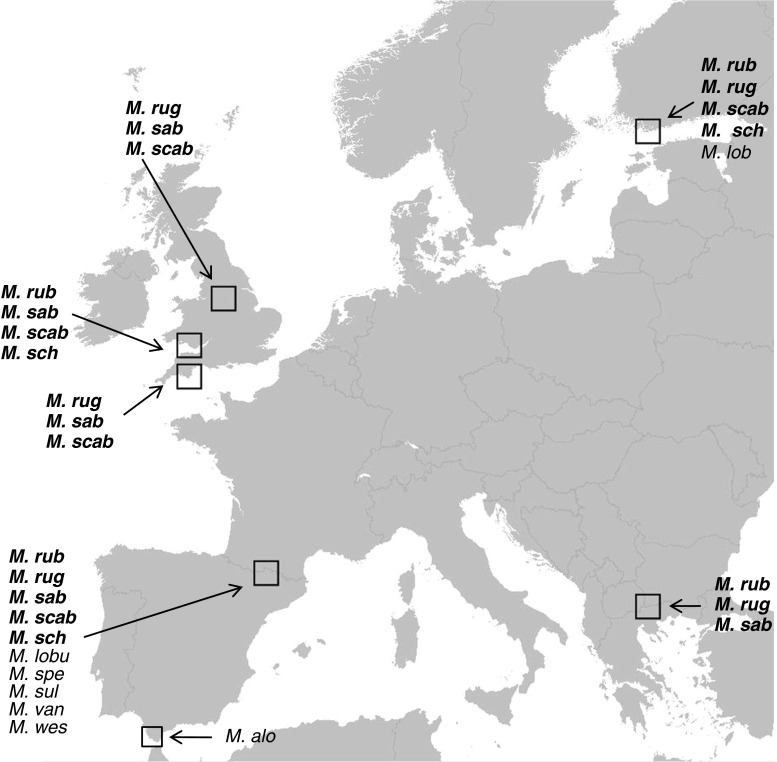
Geographic localities of the 12 *Myrmica* species collected from Great Britain, Spain, Finland, and Greece. See Online Resource 1 for locality details. rub = *M. rubra*; rug = *M. ruginodis;* sab = *M. sabuleti*; scab = *M. scabrinodis*; sch = *M. schencki*; alo = *M. aloba*; lob = *M. lobicornis*; lobu = *M. lobulicornis*; spec = *M. specioides*; sulc = *M. sulcinodis*; van = *M. vandeli*; wes = *M. wesmaeli*. Species in bold were used in a more detailed statistical analysis

This study analyzed the CHC profiles of 12 species of *Myrmica* from across Great Britain, Spain, Finland, and Greece, across 219 colonies (Fig.1; Table 1; Online Resource 1). Five species of *Myrmica* are represented from multiple countries (see Table 1), so these were used in a detailed statistical analysis to test for species-specific CHC stability. The sampling localities represent a good range of habitats and climates, and within Europe cover some of the most northerly (Finland), southerly (Spain and Greece), western (Great Britain, Spain), and eastern (Finland, Greece) distributions. Ants were collected over a period of two years from 2010 to 2012, from a number of localities within each country (Table 1, Online Resource 1). The localities in Greece and Spain were chosen as they represent two different glacial refugia - the Balkans and Iberia, respectively - thus maximizing any potential chemical diversity within species profiles. We used previous data from Guillem et al. (2012) for *M. sabuleti* and *M. scabrinodis* from Great Britain, but included an additional six colonies of *M. scabrinodis*. All samples were morphologically identified prior to chemical analysis, using keys in Radchenko and Elmes (2010). Where possible, we selected colonies from as many different sites/localities for maximum CHC diversity.

**Table 1:** *Myrmica* species, habitat type and number of colonies analyzed from each country

Species	Country	No. sites	No. colonies	Habitat	Total no. colonies
*M. aloba*	Spain	1	5	Dense & moist riverine forest.	5
*M. lobicornis*	Finland	1	3	Coniferous forest with ground moss.	3
*M. lobulicornis*	Spain	3	5	Alpine meadow above 1500 m.	5
***M. rubra***	Greece	1	2	Alder carr next to stream.	26
	Finland	5	11	Grassy fields, grass verges, inland sand dunes.	
	Spain	4	10	Grazed fields, grassy verges, alpine meadow with stream, riverine edges.	
	UK	1	3	Grassy fields.	
***M. ruginodis***	Greece	1	4	Mountainous wet glade.	26
	Finland	6	7	Coniferous forest, grassy fields, sphagnum bog.	
	Spain	10	10	Alpine meadow, glade, open pine woodland, wet meadow.	
	UK	4	5	Upland and lowland moor, limestone quarry.	
***M. sabuleti***	Greece	6	18	Rocky alpine slope, deciduous woodland edges, wet glade.	48
	Spain	10	18	Alpine meadow, forest glades, mixed forest, wet meadow.	
	UK	4	12*	Lowland moor, grassy fields, coastal sand dunes, limestone quarry.	
***M. scabrinodis***	Finland	9	25	Open pine forest, coastal grass margins, grassy fields, sphagnum bog.	63
	Spain	13	20	Alpine meadow, forest glade, open pine forest, riverside mosaic.	
	UK	7	18*	Grassy fields, upland & lowland moor, limestone quarry, coastal sand dunes	
***M. schencki***	Finland	4	8	Open pine forest, coastal grass margins, open coniferous forest.	24
	Spain	7	11	Grassy dry meadows, stony open Mediterranean scrub.	
	UK	1	5	Coastal sand dunes.	
*M. specioides*	Spain	8	8	Mosaic of meadow and woodland, dry sun exposed stony slopes, grazed alpine meadows.	8
*M. sulcinodis*	Spain	2	5	Alpine meadows above 2000 m.	5
*M. vandeli*	Spain	1	1	Alpine meadow with boggy areas, 1800 m.	1
*M. wesmaeli*	Spain	4	5	Alpine meadow with boggy areas, forest glades, 1200-1800 m.	5

*Data used from Guillem et al. (2012). Number of colonies represent the total number of nests chemo-typed. Species in bold were used in a detailed NMDS analysis

### Chemical Analyses

Chemical analyses were conducted on one sample from each colony, comprising a pooled sample of five workers (non-callows). The five ants were placed in a glass vial and immersed in 50 μl of high-performance liquid chromatography grade hexane that contained 1 mg 100 ml^−1^ of an internal standard (docosane, C_20_ alkane) for 10 min. Ants were removed, and hexane evaporated to dryness. Prior to analysis, 30 μl of hexane were added to the vials. Samples were analyzed on an HP 6890 gas chromatograph (GC) connected to an HP 5973 MSD (quadrupole) mass spectrometer (MS: −70 eV, electron impact ionization). The GC was equipped with an HP-5MS column (length 30 m; ID 0.25 mm; film thickness 0.25 μm), and the oven temperature was programmed from 70 °C to 200 °C at 40 °C min^−1^ and then from 200 °C to 380 °C at 25 °C min^−1^. Samples were injected in splitless mode, with helium as the carrier gas, at a constant flow rate of 1.0 ml min^−1^. CHCs were identified by their mass spectra, diagnostic ions, and corroborated by Kovats indices (Carlson et al. 1998). Ten extracts, three for *M. sabuleti*, three for *M. scabrinodis*, and four for *M. ruginodis* were subjected to dimethyl disulphide (DMDS) derivatization in order to determine the alkene double bond positions within the alkenes (Carlson et al. 1989). These were then re-analyzed on the GC-MS under the same conditions as the non-derivatized samples. Each DMDS sample comprised a pool of 15–20 individual ants from each country.

For each sample, the CHC abundance of each compound was determined (area under the peak) and converted to relative abundances by dividing by the total abundance in each sample. Hydrocarbons with chain lengths less than C_21_ were excluded from the analysis to avoid possible contamination of volatile compounds (i.e., pheromones) that may have derived from the Dufour’s gland. We analyzed two sets of data; first using the relative compound abundances and second using only binary data for the presence and absence of compounds. Compounds consistently less than 0.5 % between individual samples were excluded from the analysis in order to reduce the expansive dataset. Any non-hydrocarbons and contaminants were also excluded. In some cases, a small number of compounds co-eluted and it was not possible to estimate the amount of each compound, so in these cases the CHC groupings were treated as a single compound (Online Resource 2).

### Statistical Analysis

Once we had established that the 12 species each had their own species-specific CHC profile, we chose the five species with the widest geographical distribution for a more detailed analysis (*M. rubra*, *M. ruginodis*, *M. sabuleti*, *M. scabrinodis*, and *M. schencki*). Non-metric multidimensional scaling ordination plots (NMDS) were used to visualize the relative abundance and presence/absence of CHCs in the five species calculated with R 3.0.2 (R Core Team 2013) using the vegan package (Oksanen et al. 2013). NMDS uses rank values providing a more flexible technique, which accepts any form of data, omitting many of the assumptions associated with other multidimensional methods (McCune and Grace 2002). Additionally, when using large data sets, NMDS plots are visually easier to interpret than complex dendrograms produced by hierarchical clustering methods. We also plotted NMDS for all 12 species based on relative abundance data.

Prior to analysis, relative abundance data were transformed with arcsine square root to reduce the range of data values, and the NMDS was computed from Bray-Curtis distances as the dissimilarity measure. The presence/absence data were evaluated based on Jaccard distances, as these data are binary. Goodness of fit was measured with Kruskal’s STRESS (Standardized residual sum of squares). The lower the STRESS value, the better the data are represented, i.e., values of <0.05 are considered excellent, where values of >0.2 are considered a poor representation of the data and the ordination should be interpreted with caution. In addition, we calculated the average pairwise Bray-Curtis similarities based on the relative abundance of CHCs between each species by converting the dissimilarity distances as follows:

$$ {d}^{BCS}=\left(1-{d}^{BCD}\right)*100 $$

where *d* ^BCD^ is the distance measure of the Bray-Curtis dissimilarity and *d* ^BCS^ the calculated Bray-Curtis similarity measure. Therefore, a value of 1 would indicate a complete matching of the two data points in the *n*-dimensional Euclidean space, whereas zero would indicate complete separation.

## Results

**Fig. 2:**
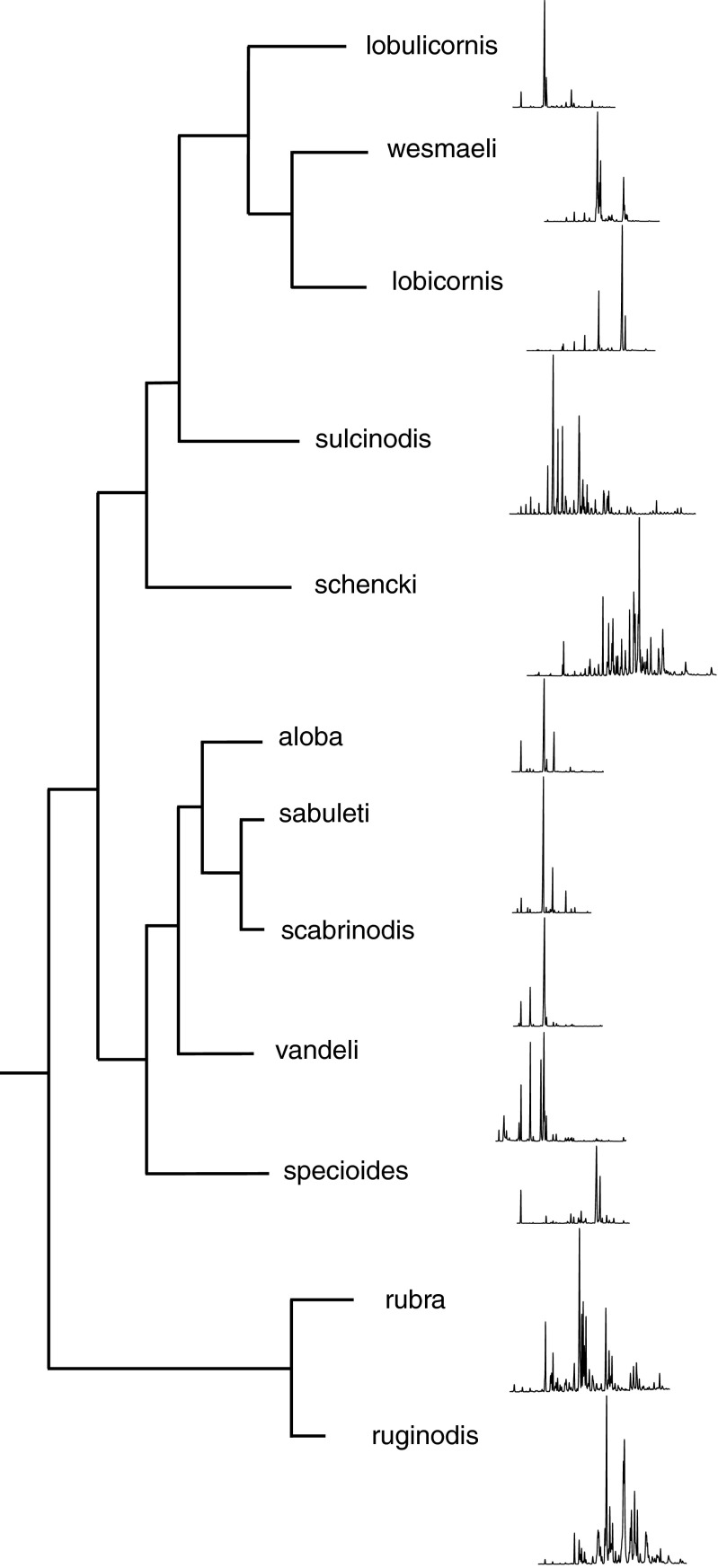
Cuticular hydrocarbon (CHC) profiles of the 12 *Myrmica* species. Chromatograms were aligned to each other using the *n*- alkanes. A simple cladogram of the 12 species is shown on the left to demonstrate genetic relatedness (based on Jansen et al. 2010). Observe how the chain lengths of the profiles shift with *M. sabuleti* and *M. scabrinodis* at the lower end (C_23_-C_29_), and *M. ruginodis* and *M. schencki* at the higher end (C_27_-C_39_), in addition to the different compounds represented by different peaks. CHC profiles shown are from Spanish populations other than *M. lobicornis* which is from Finland

**Fig. 3:**
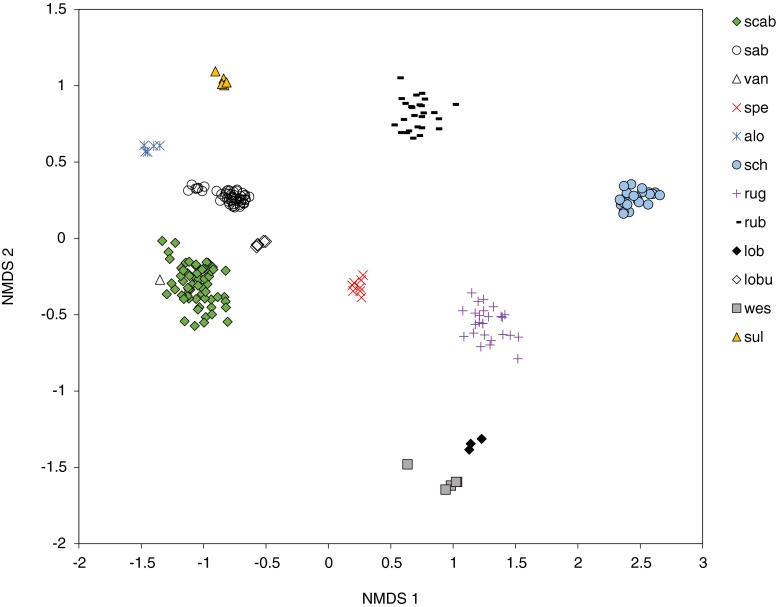
Non-metric multidimensional scaling ordination (NMDS) plot of the transformed relative proportions of compounds for 12 species of *Myrmica* based on the Bray-Curtis dissimilarity distances. STRESS = 0.075. alo = *M. aloba* (*N* = 5); lob = *M. lobicornis* (*N* = 3); lobu = *M.* lobulicornis (*N* = 5); spe = *M. specioides* (*N* = 8); sul = *M. sulcinodis* (*N* = 5); van = *M. vandeli* (*N* = 1); wes = *M. wesmaeli* (*N* = 5); rub = *M. rubra* (*N* = 26); rug = *M. ruginodis* (*N* = 26); sab = *M. sabuleti* (*N* = 48); scab = *M. scabrinodis* (*N* = 63); sch = *M. schencki* (*N* = 24)

**Fig. 4:**
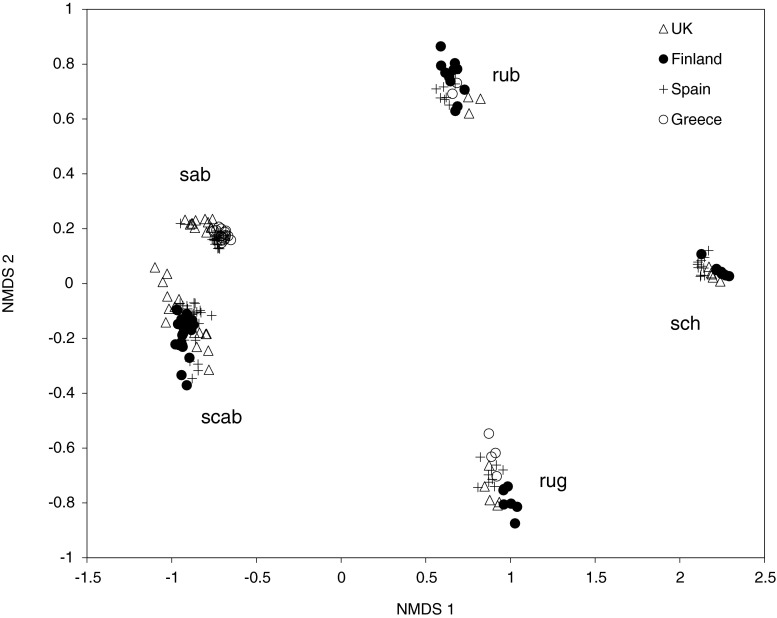
Non-metric multidimensional scaling ordination (NMDS) plot of the transformed relative proportions of compounds for five species of *Myrmica* based on the Bray-Curtis dissimilarity distances. STRESS = 0.0402. rub = *M. rubra* (*N* = 26); rug = *M. ruginodis* (*N* = 26); sab = *M. sabuleti* (*N* = 48); scab = *M. scabrinodis* (*N* = 63); sch = *M. schencki* (*N* = 24)

**Fig. 5:**
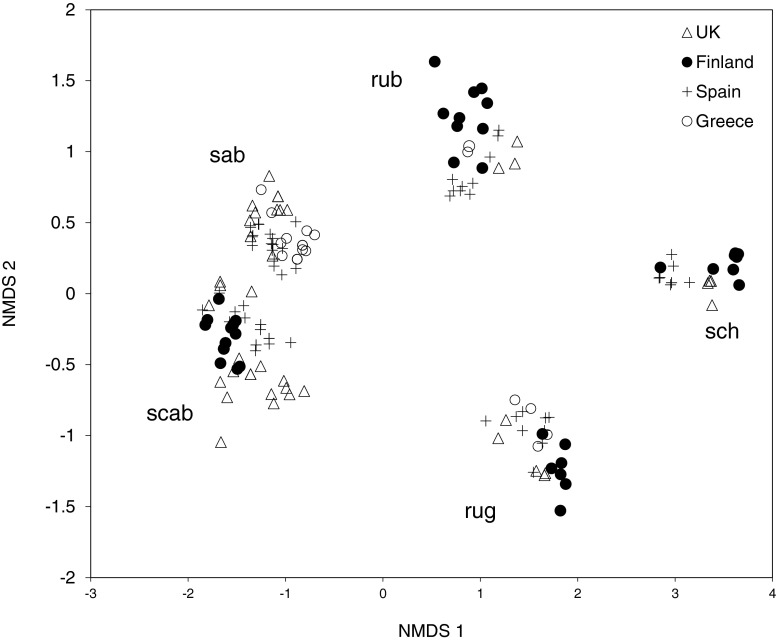
Non-metric multidimensional scaling ordination (NMDS) plot based on binary data of presence/absence of compounds for five species of *Myrmica* based on Jaccard distance. STRESS = 0.054. rub = *M. rubra* (*N* = 26); rug = *M. ruginodis* (*N* = 26); sab = *M. sabuleti* (*N* = 48); scab = *M. scabrinodis* (*N* = 63); sch = *M. schencki* (*N* = 24)

In all, 219 ant samples (one sample per colony) were analyzed, resulting in 222 hydrocarbons across the 12 study species of *Myrmica* (Online Resource 2). Twelve distinctive profiles were produced which matched the morphological identifications (Fig. 2). Compounds ranged from C_21_ to C_39_ within the 12 species. The NMDS for all 12 species produced good separation (STRESS =0.075; Fig. 3). The greatest variety of compounds was detected in the di-methyl alkanes. The NMDS ordinations of the 196 samples for the five species studied in greater detail (*M. rubra*, *M. ruginodis*, *M. sabuleti*, *M. scabrinodis*, *M. schencki*) produced excellent discrimination between these species based on both the relative proportions (STRESS =0.0402; Fig. 4) and the presence/absence of compounds (STRESS =0.054; Fig. 5). Closer inspection of the relative abundance NMDS for each of the five species revealed some population groupings although these were not always clear. This was not reflected in the presence/absence NMDS, suggesting that species profiles are highly qualitative. As more signal information is contained in the proportional data, we expected to see some geographical clustering within each species, which demonstrates the natural intra-specific variation in CHCs that is used in colony recognition.

### Inter-Specific Variability

Compound diversity among the 12 species was high (Online Resource 2), with only two compounds - heptacosane (*n*-C_27_) and nonacosane (*n*-C_29_) - found in all species. Table 2 summarizes the hydrocarbon classes. Mono-methyl alkanes and *n*-alkanes were ubiquitous across all species; however, those species rich in methyl-alkanes include *M. rubra*, *M. ruginodis*, *M. schencki*, and *M. sulcinodis*. Considerable proportions of di-methyl alkanes were found in, *M. rubra*, *M. ruginodis*, *M. schencki*, and *M. sulcinodis*, tri-methyl alkanes in *M. rubra*, *M. schencki*, and *M. sulcinodis*, whereas tetra-methyl alkanes were found only in *M. rubra*. In addition, we found small quantities of mono-methyl-alkadienes in *M. scabrinodis*, with *M. vandeli* producing surprisingly large amounts (19 %). Mono-methyl-alkenes were found only in *M. rubra* and *M. ruginodis*.

**Table 2:** Proportion (%) of hydrocarbon classes present in *Myrmica* spp. (scab = *M. scabrinodis*; sab = *M. sabuleti*; sch = *M. schencki*; rub = *M. rubra*; rug = *M. ruginodis*; alo = *M. aloba*; spe = *M. specioides;* lob = *M. lobicornis*; lobu = *M. lobulicornis*; wes = *M. wesmaeli;* sul = *M. sulcinodis*; van = *M. vandeli*

	scab	sab	sch	rub	rug	alo	spe	lob	lobu	wes	sul	van
mono-methyl alkanes	15.6	24.8	31.1	44.8	28.9	20.1	13.9	8.5	5.7	6.1	51.8	23.3
di-methyl alkanes	0.2	0.4	52.2	37.4	28.7	0	6.9	0.1	1.0	1.9	23.2	0
tri-methyl alkanes	0	0	6.0	7.4	0	0	0	0	0	0	0.7	0
tetra-methyl alkanes	0	0	0	1.4	0	0	0	0	0	0	0	0
n-alkanes	10.9	9.3	8.9	7.4	4.0	15.1	13.6	3.4	12.7	4.4	13.2	17.8
alkenes	54.2	63.5	0	1.4	13.3	0	28.2	27.2	79.8	31.8	2.6	37.7
dienes	18.1	0	0	0	21.8	59.6	36.9	62.4	0	54.3	0	0
mono-methyl alkenes	0	0	0	0.3	3.4	0	0	0	0	0	0	0
mono-methyl dienes	0.9	0	0	0	0	0	0	0	0	0	0	18.8
unknown	0	2.0	1.8	0	0	5.2	0	0	0	1.5	0	2.0

Olefin- (alkene and alkadiene) rich species include *M. aloba*, *M. lobicornis*, *M. lobulicornis*, *M. wesmaeli*, *M. sabuleti*, *M. scabrinodis*, *M. specioides*, and *M. vandeli*. The simplest chemical profile was displayed in *M. lobicornis* with 16 compounds (not including trace compounds), whereas *M. rubra* had the most complex profile with up to 72 recorded compounds (not including trace compounds).

The NMDS plots (Fig. 3) showed *M. lobicornis* to be most chemically similar to *M. wesmaeli*. Both are diene rich with hydrocarbons in the range of C_27_-C_31_, but *M. wesmaeli* has large amounts of nonacosadiene (C_29:2_ [36 %]) and nonacosene (C_29:1_ [26 %]), whereas *M. lobicornis* has large amounts of hentriacontadiene (C_31:2_ [60 %]). In contrast, the chemical profile of *M. lobulicornis* is very different from its two related siblings (Online Resource 2; Fig. 2), with little overlap in compounds.

Chemical profiles remained qualitatively stable within each species and locality, but varied quantitatively. For example, the groupings of *M. sabuleti* and *M. scabrinodis* within the NMDS were well defined in both the binary and proportional data (Figs. 4 and 5). These two sister species have similar chemical profiles, but, among other small compound differences, differences in the amounts of 3-methyl-tricosane (3-MeC_23_) and 5-methyl-pentacosane (5-MeC_25_) vary significantly enough to sufficiently separate them (for details see Guillem et al. 2012). These two species were on average among the most similar to each other, with 52.7 % similarity (Online Resource 3). Furthermore, the two species differ in the isomers of pentacosene, with *M. sabuleti* having predominantly (*Z*)-12-C_25:1_ and *M. scabrinodis* (*Z*)-9- C_25:1_ (Online Resource 2). Pentacosadiene (C_25:2_) also was present in *M. scabrinodis* but not *M. sabuleti*. The closely related *M. aloba* is alkadiene rich (60 %) with smaller amounts of mono-methyl-alkanes (20 %). This species is distinguishable from *M. scabrinodis* and *M. sabuleti* by the high presence of pentacosadiene (60 %) and complete absence of pentacosene, among other small differences.

The two sister species *M. rubra* and *M. ruginodis* differ in their CHC profiles and are only on average 25.5 % similar to each other (Online Resource 3), which is the reverse of *M. sabuleti* and *M. scabrinodis*. The highest chain compounds detected were present in *M. ruginodis*, ranging from C_25_-C_39_, whereas the chemical profile of *M. rubra* ranged from C_23_-C_31_. *Myrmica rubra* is rich in mono-methyl (45 %) and di-methyl alkanes (37 %), with few alkenes (1 %). In addition, tri- (7 %) and tetra-methyl alkanes (1 %) also were present. Tetra-methyl alkanes were conspicuous in *M. rubra*, making this species unique in this respect. In contrast, *M. ruginodis* is alkene (13 %) and diene (22 %) rich, with a lower proportion of mono- (29 %) and di-methyl-alkanes (29 %). The alkenes in *M. ruginodis* are isomer-rich, with five isomers detected in nonacosene (C_29:1_) and four in hentriacontene (C_31:1_) (Online Resource 2).

*Myrmica schencki* was well separated and the most chemically dissimilar species (Online Resource 3; also seen in Elmes et al. 2002). Forty-nine hydrocarbon compounds were detected between C_25_-C_33_. This species is rich in mono- (~31 %) and di-methyl alkanes (~52 %), with some tri-methyl alkanes present (~6 %) but absent in alkenes.

### Intra-Specific Variability

This analysis was performed only for the five *Myrmica* species from multiple countries. Within each species, colonies mainly differed in the quantitative abundance of compounds. For example, within *M. scabrinodis* there was a large proportional variation between just two compounds: pentacosene (C_25:1_) and pentacosadiene (C_25:2_), with a range of 3–82 % within C_25:1_ and 0–69 % within C_25:2_, which even occurred within populations (Online Resource 2). These compounds typically co-elute on the GC column producing only one peak in the chromatogram, but when combined, the relative proportions remain relatively consistent within the species. Average intra-specific population similarity was high (90–93 %) in *M. sabuleti* and slightly lower in *M. scabrinodis* (80–84 %; Online Resource 3), which no doubt was due to the high variation in relative abundance between pentacosene and pentacosadiene. However, the relative abundance of alkene isomers in *M. sabuleti* and *M. scabrinodis* remained both qualitatively and quantitatively similar between localities.

Even though the majority of intra-specific differences were due to varying proportions of compounds, some small qualitative differences were observed. This was particularly evident in *M. rubra*, with the Spanish population displaying the lowest number of compounds and the Finnish population the highest (Online Resource 2). This high variability in compound number was due to additional di- tri- and tetra-methyl alkanes found in Finnish samples. For example, only 4,8,12,16-tetraMeC_28_, 7,11,15- and 5,9,11-triMeC_29_ were present in this population. All additional compounds were present in low proportions however, typically <1 %, and it is possible that they also are present in the other populations but were not picked up by the GC-MS. Additionally, noticeable population differences within this species were due to shifts in positional isomers. For example, 5,9-diMeC_25_ was present in Finnish and Greek populations, whereas 5,11-diMeC_25_ was present in British and Spanish populations, 9,13 and 9,17-diMeC_29_ were present in Greek populations, whereas 9,15 and 9,17-diMe C_29_ were found in Finnish and Spanish populations. Once again, these differences in isomeric positions were <1 % relative abundance. Within *M. ruginodis*, differences mainly occurred within the alkenes, dienes, and methyl-alkadienes, with isomers of nonacosene and hentriacontene showing some quantitative variability.

Within *M. schencki*, some population differences were evident, for example the expression of 3,7-diMeC_27_ in Spanish samples but the absence of 8,14-diMeC_34_ and 9,21-diMeC_35_ from this population (Online Resource 2). These differences among others were minor and only expressed as <1 % of the total relative abundance. Again, it was mainly small quantitative differences in compounds that varied between populations.

## Discussion

This study shows that CHCs are highly species-specific and qualitatively stable between localities, despite the large geographical separation of populations. Distinct CHC profiles are displayed for each of the 12 species regardless of locality, which allows for easy discrimination among species (Fig. 2). This is despite the high diversity of habitats, ranging from coastal sand-dunes to alpine meadow, and geographically diverse climates from Mediterranean to cool continental. This stability suggests that there is a strong genetic component to CHC profiles, which is under direct selection to remain consistent within a species. Evidence for the genetic heritability of CHCs has been demonstrated in *Drosphila* (Chertemps et al. 2006, 2007; Dallerac et al. 2000) and various social insect species including ants (Drescher et al. 2010; Dronnet et al. 2006; van Zweden et al. 2009). Within the genus *Myrmica*, we showed that species can be separated reliably by the simple presence/absence of hydrocarbons alone, and proportional data are not required for taxonomic separation.

This species compound stability even extends down to the alkene isomer level, which also remained stable across localities within species. For example, there was little difference in the proportion of (*Z*)-9-pentacosene among the various populations of *M. scabrinodis* or (*Z*)-12-pentacosene in *M. sabuleti*. These isomers are a key species–specific difference between these two sister species (Guillem et al. 2012). Isomers of the more complex CHCs present within insects, such as the presence of double bonds and methyl groups, often hold the key to species signals and are more readily used in communication (Blomquist and Bagnères 2010; Martin et al. 2008a, 2008b). *Myrmica ruginodis* is unique among ants in its high occurrence of isomeric alkenes, with five detected in nonacosene and four in hentriacontene. With regards to *M. scabrinodis* and *M. ruginodis*, we propose that colony recognition cues are likely to be involved in the quantitative differences expressed within the alkenes and alkadienes. Alternatively, methyl-alkane isomers are likely to be utilized in *M. rubra* and *M. schencki*.

In contrast to *M. sabuleti* and *M. scabrinodis*, the sister species *M. rubra* and *M. ruginodis* have evolved different chemical pathways. For example, *M. rubra* is rich in mono and di-methyl alkanes, whereas *M. ruginodis* is alkene and diene rich, displaying many isomers at each chain length. This pattern of differential chemical pathways also is seen in other sister species such as *Formica fusca* and *F. lemani*, where *F. fusca* is mono and di-methyl alkane rich, and *F. lemani* is alkene rich (Martin et al. 2008a). The fact that sibling species have taken on such different chemical pathways is interesting and suggests a saltational mode of evolution (Symonds and Elgar 2008). Closely related *Tapinoma* species also strongly differ in their CHCs (Berville et al. 2013), suggesting that major changes in their chemical compositions have occurred during speciation events. Saltational shifts involve the components of a compound changing substantially or completely, and this mode of evolution results in sibling species having highly dissimilar chemical profiles, a phenomenon also seen in closely related bark beetles (Symonds and Elgar 2008) and *Yponomeuta* moths (Löfstedt et al. 1991).

Hydrocarbon diversity is generated by the insertion of methyl groups or double bonds. Rarely do both biosynthetic pathways combine to produce methylalkenes (methylalkanes with double bonds), and records of these compounds within ants are rare (Kather and Martin 2015). However, their presence in other species may have been missed due to the complex detection and identification methods required for such hydrocarbons. Methylalkenes were found in *M. rubra*, *M. ruginodis* (mono-methylalkenes), *M. scabrinodis*, and *M. vandeli* (methylalkadienes). Indeed, the high proportion of methylalkadienes present in *M. vandeli* is curious, but is based on one colony only. The NMDS plots of all 12 species show that the chemical profile of *M. vandeli* is most similar to *M. scabrinodis*. This is not surprising given the life history of *M. vandeli*, which is considered an occasional facultative social parasite of *M. scabrinodis*. In our case, the colony of *M. vandeli* found was free living, but mixed colonies of both species also have been reported (Radchenko and Elmes 2010), although rarely. It is known that social parasites mimic the chemical profile of their host colony (Guillem et al. 2014; Kleeberg and Foitzik 2015). *Myrmica vandeli* appears to have its own species specific chemical profile (i.e., a disproportionally high presence of MeC_25:2_ compared to *M. scabrinodis*) but its chemical similarity may allow it the opportunity to adapt easily its profile to that of *M. scabrinodis* when necessary. Certainly, parasitic colonies are able to up or down-regulate certain hydrocarbons thus ‘blending in’ with the surrounding host colony (Guillem et al. 2014).

The chemical profile of *M. aloba* also is of interest, displaying some features of both *M. scabrinodis* and *M. sabuleti*, appearing to sit in-between the two. For example, *M. aloba* displays a high proportion of pentacosadiene akin to *M. scabrinodis*, but a similar proportion of 5-methyl-pentacosane akin to *M. sabuleti*. Again, these three species are closely related with *M. aloba* occupying a more southerly distribution in Western Europe, restricted mainly to Iberia. Although all three species are morphologically similar, they are readily distinguishable based on their CHC profiles alone.

Three species belonging to the *lobicornis* group within Europe – *M. lobicornis*, *M. lobulicornis*, and *M. wesmaeli* - also were analyzed. Again, all three are morphologically similar. *Myrmica wesmaeli* is an Iberian endemic, most closely related to *M. lobicornis* (Jansen et al. 2010). Although these two species are among the most chemically similar to each other, both contain distinct chemical differences, which allow for easy discrimination. In contrast, *M. lobulicornis*, previously thought to be conspecific with *M. lobicornis*, is considered a montane, subalpine sibling species of *M. lobicornis*. The two species are, however, chemically different, with little chemical overlap (Online Resource 2; Fig. 2) despite remaining morphologically similar. In fact, *M. lobulicornis* is more similar chemically to both *M. sabuleti* and *M. scabrinodis*. This is yet another example where sibling species have taken on different chemical pathways.

In addition to quantitative differences, we also found some small qualitative differences within species, where some compounds were present in certain populations and absent from others. These compound differences were all minor, however, and typically occurred at proportions <1 %, so they could potentially be present in other populations just below the detectable limit of the GC-MS. It is important to recognize this natural intra-specific variation if further investigation on population and colony differences are to be explored. Colony signals often are buried within the species signal, so having the ability to up or down regulate certain compounds gives a lot of flexibility to the system, allowing each colony to display its own unique signature or odor. For example, *M. rubra* utilizes positional isomers of the 5,x-di-methyl alkane, displaying varying combinations and proportions of 5,9-, 5,11-, 5,15-, and 5,17-. This is likely to be fundamental in their colony recognition process, since it has strong parallels to the suite of colony specific di-methyl alkanes found in *Formica fusca* (Martin et al. 2008b).

There is now mounting evidence for species-specific CHC stability in several groups of ants e.g., *Tapinoma* (Berville et al. 2013), *Tetramorium* (Steiner et al. 2002), *Formica* (Martin et al. 2008a), and *Myrmica*. Much like this study, CHC profiles were not influenced by ecological factors such as vegetation type, soil or climate.

*Myrmica rubra*, one of our study species, displays western and eastern European populations belonging to different haplogroups, that form a broad secondary contact zone in Central Europe (Leppänen et al. 2011), suggesting that *M. rubra* expanded throughout Europe from multiple refugia. The authors found that Eastern Scandinavian (Finland) haplotypes were more similar to those from the Balkans, and western and central European haplotypes were more similar to each other. Interestingly, the same pattern is observed in our *M. rubra* chemo-types, where Finnish samples are more similar to Greek, and British to Spanish (Online Resource 3). However, this holds true only for *M. rubra* as this pattern of similarity is not demonstrated in *M. ruginodis*. Eastern and western haplogroups also are evident in other species of ants such as *Formica* (Goropashnaya et al. 2004) and *Temnothorax* (Pusch et al. 2006). Parts of southern Europe, such as Iberia, have served as refugia for many taxa (Hewitt 1999), including ants (Beibl et al. 2007; Pusch et al. 2006; Schlick-Steiner et al. 2007). Based on the age of these refugia, our data raises the possibility that CHCs may have remained stable within our five study species over millennia. Thus, CHCs appear not to be under slow mutational drift, but remain stable due to a genetic heritability and selective forces, as would be expected of compounds used in recognition.

In summary, we found that species-specific CHC profiles remain remarkably stable throughout their geographical ranges and appear not to be influenced by climate, vegetation, or soil type. This stability of compounds within profiles suggests that surface chemistry is under strong genetic selection despite populations remaining isolated from each other for long periods of time. Ants use CHCs as colony and species recognition cues (Howard 1993; Lucas et al. 2005), which are buried within the typical species-signal, so the need to remain under genetic influence is crucial as even a slight change in chemical composition can lead to rapid speciation events (Roelofs et al. 2002; Roelofs, and Rooney, 2003).

## Electronic supplementary material

ESM 1 (PDF 132 kb)

ESM 2 (PDF 727 kb)

ESM 3 (DOCX 19 kb)
