# Supplementary material for: Species-Specific Cuticular Hydrocarbon Stability within European Myrmica Ants
Source: J Chem Ecol. 2016 Nov 10;42(10):1052–62. doi: 10.1007/s10886-016-0784-x (PMC5119849; doi:10.1007/s10886-016-0784-x)
Supplement: Supplementary file 1 — (PDF 132 kb) [file 10886_2016_784_MOESM1_ESM.pdf]

SPECIES-SPECIFIC CUTICULAR HYDROCARBON STABILITY WITHIN EUROPEAN *Myrmica* ANTS

RHIAN M. GUILLEM [1, 2,] *, FALKO P. DRIJFHOUT [3], STEPHEN J. MARTIN [4]

[1]*Department of Animal and Plant Sciences, University of Sheffield, Sheffield S10 2TN, UK*

[2]*Department of Life & Earth Sciences, Gibraltar Botanic Gardens Campus, University of Gibraltar, Gibraltar*

[3]*Chemical Ecology Group, School of Physical and Geographical Sciences, Lennard-Jones Laboratory, Keele University, Keele ST5 5BG, UK*

[4]*School of Environment & Life Sciences, University of Salford, Manchester M5 4WT, UK*

* Corresponding author. E-mail: rguillem@gibraltargardens.gi

Online Resource 1 Collection sites for *Myrmica* ants. Latitude and longitude are given in decimal World Geodetic System. Numbers of colonies given are those which were chemo-typed and statistically analyzed.

Date	Species	Location	Latitude	Longitude	Elevation	Country	No. colonies	Habitat
7.10.2010	M. scabrinodis	Slapton Ley	50.300833N	3.650277W	46m	England	3	Grassy, open field.
6.10.2010	M. scabrinodis	Dartmoor	50.456944N	4.060277W	213m	England	2	Lowland Moor.
6.10.2010	M. scabrinodis	Dartmoor	50.4650N	4.0825W	170m	England	4	Lowland Moor.
13.10.2010	M. scabrinodis	Fulwood Booth	53.362669N	1.586294W	365m	England	2	Upland moor with rocky outcrops.
13.10.2010	M. scabrinodis	Stoney Middleton	53.281111N	1.687777W	244m	England	2	Disused limestone quarry.
8.04.2011	M. scabrinodis	Cardiff	51.541486N	3.201375W	103m	Wales	1	Mowed open grass field.
30.5.2011	M. scabrinodis	Kenfig	51.515555N	3.741666W	9m	Wales	4	Coastal sand dunes.
18.8.2012	M. scabrinodis	Joskär Island	59.845491N	23.254722E	16m	Finland	1	Island with open *Pinus sylvestris* woodland.
19.8.2012	M. scabrinodis	Hanko	59.842033N	23.247300E	10m	Finland	1	Coastal grass margins next to reed bed.
19.8.2012	M. scabrinodis	Hanko	59.843033N	23.237333E	18m	Finland	6	Open coniferous forest with large granite outcrops. Ground moss & lichen, with boggy areas.
20.8.2012	M. scabrinodis	Hanko	59.827700N	23.015650E	23m	Finland	1	Coniferous forest with thick ground covering of moss and lichen.
20.8.2012	M. scabrinodis	Tvärminneön	59.843286N	23.224966E	8m	Finland	3	Agricultural field with long grass.
21.8.2012	M. scabrinodis	Harparskog	59.933033N	23.318883E	17m	Finland	4	Open Sphagnum bog with scattered small *Pinus* and *Betula*.
21.8.2012	M. scabrinodis	Tvärminneön	59.843983N	23.233233E	4m	Finland	3	Agricultural field with long grass
21.8.2012	M. scabrinodis	Porsgrundet Island	59.842233N	23.252516E	8m	Finland	4	Rocky, open grassy outcrops.
22.8.2012	M. scabrinodis	Tvärminneön	59.843286N	23.224966E	8m	Finland	2	Agricultural field with long grass.
18.6.2011	M. scabrinodis	Val de Ruda	42.671366N	0.956666E	1595m	Spain	1	Alpine meadow with stream.
18.6.2011	M. scabrinodis	Mata de Valencia	42.639233N	1.028350E	1574m	Spain	2	*Abies alba* forest glade.
18.6.2011	M. scabrinodis	Centre Natura Les Planes de Son	42.618133N	1.082000E	1536m	Spain	1	Alpine meadow.
19.6.2011	M. scabrinodis	3.4km from Son	42.617583N	1.054450E	1902m	Spain	2	Open woodland of *Pinus nigra* alongside small glades with several streams.
20.6.2011	M. scabrinodis	Val de Varradòs	42.749100N	0.761733E	949m	Spain	2	Flat, wet meadow next to stream, surrounded by forest of *Pinus sylvestris.*

Date	Species	Location	Latitude	Longitude	Elevation	Country	No. colonies	Habitat
20.6.2011	M. scabrinodis	Val de Varradòs	42.776800N	0.831966E	1560m	Spain	1	Flat, grazed meadow next to stream surrounded by forest of *Fagus sylvaticus*.
20.6.2011	M. scabrinodis	Val de Ruda	42.643466N	0.822083E	1860m	Spain	1	Rocky meadow.
22.6.2011	M. scabrinodis	Prats del Coro	42.621777N	1.071222E	1670m	Spain	2	Mosaic of meadow with bracken and *Juniperus communis* scrub.
22.6.2011	M. scabrinodis	Pas del Coro	42.623361N	1.061638E	1811m - 1850m	Spain	2	Meadow with boggy and dry areas.
23.6.2011	M. scabrinodis	Pla de Beret	42.731444N	0.982777E	2071m	Spain	1	Short, sparse alpine meadow on ski slope with scattered rocks.
27.6.2011	M. scabrinodis	Ribera de Sant Martí	42.509138N	0.876222E	1702m	Spain	2	Flat, dry meadow next to river and open *Pinus* woodland.
27.6.2011	M. scabrinodis	near Boi Taüll Ski Station	42.486055N	0.864388E	1913m	Spain	2	Alpine meadow on slope next to stream, with wet and dry areas.
27.6.2011	M. scabrinodis	near Boi Taüll Ski Station	42.502111N	0.857305E	1788m	Spain	1	Very short, flat, grazed alpine meadow.
6.10.2010	M. sabuleti	Dartmoor	50.456944N	4.060277W	213m	England	3	Lowland moor.
13.10.2010	M. sabuleti	Stoney Middleton	53.281111N	1.687777W	244m	England	3	Disused sheltered limestone quarry.
18.5.2010	M. sabuleti	Slapton Ley	50.300833N	3.650277W	46m	England	3	Open, grassy, inclined field.
30.5.2011	M. sabuleti	Kenfig	51.515555N	3.741666W	9m	Wales	3	Coastal sand dunes.
6.10.2012	M. sabuleti	top of Mount Kerkini	41.314500N	23.216166E	1560m	Greece	4	Rocky alpine slope with herbs, grasses & scattered prostrate *Juniperus.*
6.10.2012	M. sabuleti	top of Mount Kerkini	41.315783N	23.218116E	1533m	Greece	3	Rocky alpine slope with herbs, grasses & scattered prostrate *Juniperus.*
6.10.2012	M. sabuleti	Mount Kerkini	41.319166N	23.202333E	1480m	Greece	2	Wet glade on slope surrounded by *Fagus sylvaticus* forest.
9.10.2012	M. sabuleti	Mount Kerkini	41.341716N	23.234066E	1264m	Greece	1	Mixed woodland with *Fagus*, *Castanea*, & *Betula.*
9.10.2012	M. sabuleti	Mount Kerkini	41.315833N	23.213566E	1593m	Greece	5	Rocky alpine slope with herbs, grasses & scattered prostrate *Juniperus.*
9.10.2012	M. sabuleti	Mount Kerkini	41.313066N	23.213316E	1582m	Greece	3	Rocky alpine slope with grasses, bracken & herbs.
18.6.2011	M. sabuleti	Val de Ruda	42.671366N	0.956666E	1595m	Spain	5	Meadow with stream.
18.6.2011	M. sabuleti	Mata de Valencia	42.639233N	1.028350E	1574m	Spain	1	Glade on forest edge.
19.6.2011	M. sabuleti	Centre Natura Les Planes de Son	42.618133N	1.082000E	1536m	Spain	2	Alpine meadow.
20.6.2011	M. sabuleti	Val de Varradòs	42.749100N	0.761733E	949m	Spain	1	Flat, wet alpine meadow.
20.6.2011	M. sabuleti	Val de Varradòs	42.776800N	0.831966E	1560m	Spain	1	Flat, grazed meadow next to stream.
21.6.2011	M. sabuleti	near Jou	42.603116N	1.116716E	1370m	Spain	1	Alpine meadow.
21.6.2011	M. sabuleti	near Plana de la Font from Jou	42.604583N	1.101516E	1469m	Spain	2	Mixed broadleaf & conifer woodland.
22.6.2011	M. sabuleti	Prats del Coro	42.621777N	1.071222E	1670m	Spain	1	Mosaic of meadow with bracken & *Juniperus communis* scrub.
22.6.2011	M. sabuleti	Pas del Coro	42.623361N	1.061638E	1820m	Spain	3	Meadow with some boggy areas.
23.6.2011	M. sabuleti	Bosc de Bonabé	42.751805N	1.084527E	1500m	Spain	1	Mosaic of meadow next to stream.
18.6.2011	M. schencki	Val de Ruda	42.671366N	0.956666E	1595m	Spain	1	Meadow with stream.
22.6.2011	M. schencki	near Son	42.633361N	1.104444E	1193m	Spain	2	Grass meadow on steep slope.
22.6.2011	M. schencki	Pas del Coro	42.623361N	1.061638E	1820m	Spain	1	Meadow with some boggy areas.
22.6.2011	M. schencki	Esterri d'Aneu	42.623388N	1.125333E	965m	Spain	4	Flat field left fallow for grazing horses.
24.6.2011	M. schencki	near Isil	42.693083N	1.096305E	1255m	Spain	1	Stony, open & dry slope with Mediterranean flora.
27.6.2011	M. schencki	near Taüll	42.511138N	0.862750E	1658m	Spain	1	Open scrub of *Cytisus purgans* on mountain slope.
27.6.2011	M. schencki	Ribera de Sant Martí	42.509138N	0.876222E	1702m	Spain	1	Flat, dry meadow next to river.
18.8.2012	M. schencki	Joskär Island	59.845491N	23.254722E	16m	Finland	3	Open forest of *Pinus sylvestris.*
19.8.2012	M. schencki	Hanko	59.842033N	23.247300E	10m	Finland	1	Coastal grass margins next to reed bed.
19.8.2012	M. schencki	Hanko	59.843033N	23.237333E	18m	Finland	2	Open coniferous forest with large granite outcrops. Ground moss & lichen with boggy areas.
20.8.2012	M. schencki	Hanko	59.827700N	23.015650E	23m	Finland	2	Coniferous forest with thick ground covering of moss and lichen.
7.9.2012	M. schencki	Merthyr Mawr	51.475383N	3.639516W	12m	Wales	5	Successional coastal sand dunes
20.8.2012	M. rubra	Hanko	59.845116N	23.207700E	13m	Finland	2	Inland sand dunes surrounded by *Pinus forest*. Ground covering of moss and lichen.
21.8.2012	M. rubra	Porsgrundet Island	59.842233N	23.252516E	8m	Finland	4	Rocky, open grassy outcrops.
22.8.2012	M. rubra	Tvärminneön	59.843286N	23.224966E	8m	Finland	2	Agricultural field with long grass.
22.8.2012	M. rubra	Lappohja train station	59.904138N	23.236741E	28m	Finland	3	Grassy verges.

Date	Species	Location	Latitude	Longitude	Elevation	Country	No. colonies	Habitat
7.10.2012	M. rubra	Alder carr, Mount Kerkini	41.319166N	23.202333E	62m	Greece	2	Alder carr next to stream.
24.6.2011	M. rubra	Esterri d'Aneu	42.618611N	1.125555E	950m	Spain	3	Grazed field and road margins
25.6.2011	M. rubra	near Sarroca de Bellera	42.358777N	0.878277E	933m	Spain	3	Flat meadow and stream with riverine deciduous woodland.
27.6.2011	M. rubra	towards Boi Taüll Ski Station	42.486055N	0.864388E	1913m	Spain	1	Alpine meadow on slope next to stream.
28.6.2011	M. rubra	Barruera	42.500277N	0.800277E	1090m	Spain	3	Mosaic of riverside, wooded garden edges and fairly dry flat meadow
8.4.2011	M. rubra	Cardiff	51.541486N	3.201375W	103m	Wales	3	open grass mowed field
19.8.2012	M. ruginodis	Hanko	59.842033N	23.247300E	10m	Finland	1	Open coniferous forest with large granite outcrops. Ground moss & lichen with boggy areas.
20.8.2012	M. ruginodis	Hanko	59.827700N	23.015650E	23m	Finland	1	Coniferous forest with thick ground covering of moss and lichen.
20.8.2012	M. ruginodis	Tvärminneön	59.843286N	23.224966E	8m	Finland	1	Agricultural field with long grass.
21.8.2012	M. ruginodis	Harparskog	59.933033N	23.318883E	17m	Finland	2	Open bog with Sphagnum. Scattered small *Pinus* and *Betula*.
21.8.2012	M. ruginodis	Tvärminneön	59.843983N	23.233233E	4m	Finland	1	Agricultural field with long grass.
21.8.2012	M. ruginodis	Porsgrundet Island	59.842233N	23.252516E	8m	Finland	1	Rocky, open grassy outcrops.
6.10.2012	M. ruginodis	Mount Kerkini	41.319166N	23.202333E	1480m	Greece	4	Glade on slope with some wet areas surrounded by forest of *Fagus sylvaticus*.
18.6.2011	M. ruginodis	Mata de Valencia	42.639233N	1.028350E	1574m	Spain	1	Glade on edge of *Abies alba* forest.
19.6.2011	M. ruginodis	Centre Natura Les Planes de Son	42.618133N	1.082000E	1536m	Spain	1	Alpine meadow.
19.6.2011	M. ruginodis	3.4km from Son	42.617583N	1.054450E	1902m	Spain	1	Open woodland of *Pinus nigra* alongside small glades with several streams.
20.6.2011	M. ruginodis	Val de Varradòs	42.749100N	0.761733E	949m	Spain	1	Flat, wet meadow next to stream.
20.6.2011	M. ruginodis	Val de Ruda	42.643466N	0.822083E	1860m	Spain	1	Rocky meadow.
21.6.2012	M. ruginodis	near Jou	42.603116N	1.116716E	1370m	Spain	1	Alpine meadow on slope.
21.6.2011	M. ruginodis	Towards Plana de la Font from Jou	42.604583N	1.101516E	1469m	Spain	1	Mixed broadleaf & conifer woodland.
22.6.2011	M. ruginodis	near Son	42.633361N	1.104444E	1193m	Spain	1	Meadow on steep slope.
22.6.2011	M. ruginodis	Pas del Coro	42.623361N	1.061638E	1820m	Spain	1	Meadow with some boggy areas.
23.6.2011	M. ruginodis	Bosc de Bonabé	42.751805N	1.084527E	1500m	Spain	1	Mosaic of meadow next to stream.
21.6.2010	M. ruginodis	Surprise View	53.317777N	1.626388W	318m	England	1	Moorland with wooded areas.
6.10.2010	M. ruginodis	Dartmoor	50.456944N	4.060277W	213m	England	1	Lowland moor.
13.10.2010	M. ruginodis	Fulwood Booth Farm	53.362669N	1.586294W	365m	England	2	Upland moor with rocky outcrops.
13.10.2010	M. ruginodis	Stoney Middleton	53.281111N	1.687777W	244m	England	1	Disused limestone quarry.
13.07.2011	M. aloba	Montera del Torero, Cadiz	36.224967N	5.583917W	120m	Spain	5	Dense and moist riverine forest of *Quercus suber*, *Q. canariensis* & *Alnus glutinous*.
19.08.2012	M. lobicornis	Hanko	59.843033N	23.237333E	18m	Finland	3	Open forest of *Pinus sylvestris*, *Picea abies*, & *Betula* sp. with large rocky granite outcrops, ground moss and lichen, boggy areas.
18.06.2011	M. lobulicornis	Port de la Bonaigua	42.6640667N	0.9807833E	2075m	Spain	1	Alpine meadow.
21.06.2011	M. lobulicornis	Refugi de la Plana de la Font	42.5954333N	1.0744833E	2070m	Spain	3	Alpine meadow on slope, bordered with woodland of *Pinus nigra*.
23.06.2011	M. lobulicornis	Bosc de Bonabé	42.7518056N	1.0845278E	1500m	Spain	1	Mosaic of meadow and forest edge with *Betula* & *Abies*, next to stream.
19.06.2011	M. specioides	Centre Natura Les Planes de Son	42.618133N	1.081E	1536m	Spain	1	Mosaic of meadow and woodland with *Corylus avellana*, *Pinus nigra*, *Juniperus communis*, *Betula pendula* and *Rosa* sp.
21.06.2011	M. specioides	near Jou	42.603117N	1.116717E	1370m	Spain	1	Meadow on slope with scattered *Rosa* sp., *Juniperus communis* & *Pinus nigra*.
22.06.2011	M. specioides	near Son	38.001667N	1.104444E	1193m	Spain	1	Meadow on steep slope and surrounding woodland of *Quercus humilis*, *Fraxinus excelsior*, *Sorbus acuparia*, *Corylus avellana* & *Fagus sylvaticus*.
23.06.2011	M. specioides	near Isil	41.585N	1.096111E	1255m	Spain	1	Stony, open, sunny and dry slope with scattered *Rubus* sp. & *Rosa* sp.

Date	Species	Location	Latitude	Longitude	Elevation	Country	No. colonies	Habitat
25.06.2011	M. specioides	near Sarroca de Bellera	21.526667N	0.878056E	933m	Spain	1	Flat meadow and stream with riverine woodland of *Fagus sylvatica*, *Populus nigra*, *Corylus avellana* and *Crataegus monogyna*.
26.06.2011	M. specioides	near Els Castells	18.221667N	1.173889E	1630m	Spain	1	Flat mountain meadow.
27.06.2011	M. specioides	towards Boi Taüll Ski Station	30.126667N	0.857222E	1788m	Spain	1	Very short, flat, grazed alpine meadow.
28.06.2011	M. specioides	Barruera	42.500278N	0.800278E	1090m	Spain	1	Mosaic of riverside, wooded garden edges and fairly dry flat meadow.
18.06.2011	M. sulcinodis	Port de la Bonaigua	42.664067N	0.980783E	2075m	Spain	3	Alpine meadow with flatter areas and some ponds.
21.06.2011	M. sulcinodis	Refugi de la Plana de la Font	42.595433N	1.074483E	2070m	Spain	2	Alpine meadow on slope, bordered with woodland of *Pinus nigra.*
22.06.2011	M. vandeli	Pas del Coro	42.6233611N	1.0616389E	1811m	Spain	1	Meadow with boggy and dry areas, surrounded by forest of *Pinus nigra*, *Abies alba* and scrub of *Cytisus purgans* along forest edges.
18.06.2011	M. wesmaeli	Mata de Valencia	42.639233N	1.02835E	1574m	Spain	1	Glade on edge of forest and interior of forest with *Abies alba*, *Betula pendula* & *Corylus avellana*.
21.06.2011	M. wesmaeli	Near Jou	42.603117N	1.116717E	1370m	Spain	1	Meadow on slope with scattered *Rosa* sp., *Juniperus communis* & *Pinus nigra*.
22.06.2011	M. wesmaeli	Near Son	38.001667N	1.104444E	1193m	Spain	2	Meadow on steep slope and surrounding woodland of *Quercus humilis*, *Fraxinus excelsior*, *Sorbus acuparia*, *Corylus avellana* & *Fagus sylvaticus.*
22.06.2011	M. wesmaeli	Pas del Coro	37.401667N	1.061389E	1811m	Spain	1	Meadow with boggy and dry areas, surrounded by forest of *Pinus nigra*, *Abies alba* and scrub of *Cytisus purgans* along forest edges.
