# Supplementary material for: Species-Specific Cuticular Hydrocarbon Stability within European Myrmica Ants
Source: J Chem Ecol. 2016 Nov 10;42(10):1052–62. doi: 10.1007/s10886-016-0784-x (PMC5119849; doi:10.1007/s10886-016-0784-x)
Supplement: Supplementary file 2 — (PDF 727 kb) [file 10886_2016_784_MOESM2_ESM.pdf]

Online Resource 2 Mean relative proportions % ± s.d for populations of *Myrmica* (*M. scabrinodis* (scab), *M. sabuleti* (sab), *M. schencki* (sch), *M. rubra* (rub) and *M. ruginodis* (rug) from Finland (F), Spain (S), Britain (UK), and Greece (G). van = *M. vandeli*, spe = *M. specioides*, lob = *M. lobicornis*, lobu = *M. lobulicornis*, wes = *M. wesmaeli*, sul = *M. sulcinodis*, alo = *M. aloba*. RI = Retention Index. RI's were calculated relative to alkane standards based on van Den Dool and Kratz (1963). The Retention Indices of some compounds could not be calculated as no reference alkanes were available and these are indicated as –; *t* refers to trace compound <0.2%. * = 1 isomer, ** = 2 isomers, *** = 3 isomers present within a compound. [a] = consists of a number of undetermined isomers based on RI. Compounds *in parentheses* consist of a minor component of that MS peak.

Compound	RI	scabUK	scabF	scabS	sabUK	sabS	sabG	schUK	schF	schS	rubUK	rubF	rubS	rubG	rugUK	rugF	rugS	rugG	van	spe	lob	lobu	wes	sul	alo
C21	2100																		1.63						
3-MeC21	2173																		2.31						
C22	2200	*t*	*t*	*t*															0.64	*t*				0.29 ± 0.10	
MeC23:2	2261																		*t*						
3-MeC22	2271	*t*	*t*	*t*															0.60						
C23:2	2271																								*t*
C23:1(all isomers)	2275-2279	0.90 ± 0.34 *	0.61 ± 0.12 *	1.34 ± 0.35	1.06 ± 0.82 *	1.42 ± 0.47 *	0.57 ± 0.21 *												2.93 *	*t* *		0.6 ± 0.08 ***		0.20 ± 0.13*	*t* *
n-C23	2300	5.16 ± 2.52	7.3 ± 1.9	9.86 ± 2.04	4.87 ± 1.47	7.05 ± 1.31	4.33 ± 0.98				0.40 ± 0.27	0.17 ± 0.1	0.58 ± 0.35	*t*					9.43	7.94 ± 2.04		6.62 ± 1.05	*t*	5.04 ± 1.21	9.78 ± 1.68
9-;11-;13-MeC23	2336	*t*	*t*	*t*	0.52 ± 0.73	0.21 ± 0.06	0.17 ± 0.06													*t*				2.91 ± 0.73	
7-MeC23	2341	*t*	*t*	*t*	*t*	*t*	*t*													*t*					0.34 ± 0.06
5-MeC23	2351	*t*	*t*	*t*	1.66 ± 0.49	3.2 ± 1.37	3.14 ± 0.89				0.33 ± 0.13	0.33 ± 0.17	0.43 ± 0.28	0.16 ± 0.03						*t*				*t*	1.70 ± 0.57
unknown	2358																		0.3						
4-MeC23	2361	*t*	*t*	*t*															*t*	*t*					
3-MeC23	2374	11.08 ± 2.63	15.96 ± 2.84	12.76 ± 3.39	0.65 ± 0.48	1.74 ± 1.13	0.70 ± 0.41												18.70	*t*		*t*		12.03 ± 1.23	1.28 ± 0.11
C24:1 [a]	2371-2394	0.3 ± 0.3	0.02 ± 0.08	0.79 ± 0.26	0.65 ± 0.26	0.55 ± 0.21	0.67 ± 0.12												0.36			0.79 ± 0.05 **			*t*
n-C24	2400	0.27 ± 0.11	0.45 ± 0.14	0.81 ± 0.21	0.25 ± 0.25	0.30 ± 0.06	0.14 ± 0.02												0.77	0.21 ± 0.43		0.4 ± 0.07		1.77 ± 0.55	0.81 ± 0.31
3,13-diMeC23	2409																							3.87 ± 1.60	
3,7-diMeC23	2410	*t*	*t*	*t*	*t*	*t*	*t*												*t*				0.30 ± 0.26		
8-;11-;12-;14-MeC24	2435																							4.10 ± 0.64	

Compound	RI	scabUK	scabF	scabS	sabUK	sabS	sabG	schUK	schF	schS	rubUK	rubF	rubS	rubG	rugUK	rugF	rugS	rugG	van	spe	lob	lobu	wes	sul	alo
5-MeC24	2452				0.37 ± 0.19	0.34 ± 0.08	0.17 ± 0.13																		0.28 ± 0.04
4-MeC24	2460	*t*		*t*																*t*				*t*	0.20 ± 0.04
MeC25:2 [a]	2443-2463	1.01 ± 0.85	*t*	*t*															16.76						
3-MeC24+C25:2	2473																							1.18 ± 0.29	
C25:2 [a]	2465-2489	6.85 ± 12.25	23.3 ± 20.91	19.78 ± 22.82																					57.98 ± 5.67
C25:1 (all isomers)	2472-2497	66.57 ± 16.97	45.62 ± 19.22	44.24 ± 22.37	59.77 ± 3.58	47.24 ± 3.48	49.96 ± 4.66	*t*	*t*	*t*	0.14 ± 0.12	0.51 ± 0.27	0.91 ± 0.58	0.76 ± 0.35	*t*	*t*	*t*	*t*	31.37**	0.47 ± 0.25 **		69.01 ± 2.39 **	0.27 ± 0.59	1.68 ± 0.77	
(*Z*)-12-C25:1	2472	3.11			94.05	96.01	94.89																		
(*Z*)-11-C25:1	2479				1.4	3.45	4.86																		
(*Z*)-9-C25:1	2478	96.89	98.68	98.37	4.55	0.54	0.25																		
(*Z*)-7-C25:1	2483		1.32	1.63																					
4,10-diMeC24	2493																							0.32 ± 0.08	
unknown	2494																								2.18 ± 2.10
n-C25	2500	1.66 ± 0.84	2.3 ± 0.52	4.36 ± 1.06	2.79 ± 1.22	4.79 ± 1.3	3.13 ± 0.55				3.1 ± 2.21	2.98 ± 1.3	6.09 ± 2.82	3.48 ± 1.5	0.36 ± 0.16	*t*	0.35 ± 0.13	0.38 ± 0.32	4.48	1.97 ± 0.56		3.88 ± 0.77	0.3 ± 0.1	5.48 ± 2.36	3.49 ± 2.54
3,11-diMeC24	2507																							0.26 ± 0.03	
unknown	2509																								0.48 ± 0.14
unknown	2520				0.47 ± 0.14	0.57 ± 0.17	1.02 ± 0.25																		
9-;11-;13-MeC25	2534	*t*	*t*	*t*	1.57 ± 0.53	2.01 ± 0.52	2.47 ± 0.57				1.43 ± 0.29	2.49 ± 1.0	4.21 ± 1.21	1.24 ± 0.01					0.22	0.23 ± 0.13	*t*	1.03 ± 0.27		23.34 ± 1.26	0.21 ± 0.13
7-MeC25	2542	*t*	*t*	*t*	0.70 ± 0.34	0.39 ± 0.23	0.28 ± 0.06				1.28 ± 0.4	1.44 ± 1.14	2.11 ± 0.6	1.12 ± 0.08						*t*					
unknown	2550																		1.39						
5-MeC25	2552	0.72 ± 0.65	0.44 ± 0.25	1.37 ± 1.13	13.67 ± 2.17	18.27 ± 2.64	12.7 ± 2.9				2.35 ± 0.47	3.6 ± 1.24	2.38 ± 0.65	2.66 ± 0.02			*t*	*t*		0.87 ± 0.37		0.69 ± 0.25		0.64 ± 0.07	15.28 ± 1.73
4-MeC25	2562		*t*	*t*																*t*					
11,15+9,13-diMeC25	2565												0.42 ± 0.3												
9,13-diMeC25	2567										0.48 ± 0.14	0.74 ± 0.4	0.07 ± 0.16	0.51 ± 0.01											
9,13+9,15-diMeC25	2569																							1.92 ± 0.40	
C26:1 [a]	2569-2577				0.81 ± 0.26		1.05 ± 0.16															0.53 ± 0.08			

Compound	RI	scabUK	scabF	scabS	sabUK	sabS	sabG	schUK	schF	schS	rubUK	rubF	rubS	rubG	rugUK	rugF	rugS	rugG	van	spe	lob	lobu	wes	sul	alo
7,11+7,15-diMeC25 (+3-MeC25)	2574										0.86 ± 0.21	2.12 ± 0.93	1.49 ± 0.36	1.06 ± 0.04											
3-MeC25	2574	0.57 ± 0.22	1.53 ± 0.6	1.54 ± 0.58	0.60 ± 0.39	1.9 ± 0.34	0.84 ± 0.53												1.26	0.43 ± 0.18		0.987 ± 0.18	*t*	7.69 ± 0.79	0.71 ± 0.71
5,9-diMeC25	2583	*t*	*t*	*t*								0.36 ± 0.15		0.29 ± 0.02											0.33 ± 0.03
5,11-diMeC25	2583										0.97 ± 0.06		1.19 ± 0.4												
5,15-diMeC25	2583																							0.65 ± 0.07	
5,17-diMeC25	2584	*t*	*t*	*t*																					*t*
n-C26	2600	*t*	*t*	*t*		*t*	*t*				0.34 ± 0.2	0.26 ± 0.09	0.57 ± 0.23	0.42 ± 0.22					*t*	*t*		0.24 ± 0.15	0.25 ± 0.42		*t*
7,11,15-triMeC25	2601																							0.72 ± 0.1	
3,13-diMeC25	2609				0.42 ± 0.17	0.33 ± 0.08	0.34 ± 0.09																	13.92 ± 1.96	
3,9-diMeC25	2609	*t*		*t*															*t*						
3,7-diMeC25	2610										0.45 ± 0.06	0.17 ± 0.16	0.48 ± 0.09									0.95 ± 0.39			
10-;12-;14-MeC26	2633										0.69 ± 0.06	0.88 ± 0.36	1.34 ± 0.14	0.47 ± 0.01				*t*		*t*				1.91 ± 0.13	
8-MeC26	2639										0.26 ± 0.08	0.44 ± 0.14	0.63 ± 0.18	0.51 ± 0.01											
3,7,11-triMeC25	2641																							0.55 ± 0.27	
6-MeC26	2646										*t*	*t*	*t*	*t*											
MeC27:2	2648	*t*																							
5-MeC26	2651				*t*	*t*	*t*				*t*	*t*	*t*	*t*				*t*							
unknown	2654																		0.44						
C27:2 [a]	2655-2677	*t*	*t*	0.29 ± 0.34																0.90 ± 0.16 **					
4-MeC26	2659					*t*					*t*	*t*	*t*	*t*											
MeC27:2	2662																		0.78						
10,14-diMeC26	2665										0.91 ± 0.17	0.35 ± 0.12	0.63 ± 0.19	0.22 ± 0.06											
8,12-diMeC26	2668										0.16 ± 0.14	0.56 ± 0.17	0.26 ± 0.15	0.47 ± 0.01										0.80 ± 0.16	
unknown	2672																								1.95 ± 0.37
6,10-diMeC26	2677										0.41 ± 0.17	0.24 ± 0.11	0.41 ± 0.2	*t*											
unknown	2688																								0.41 ± 0.06

Compound	RI	scabUK	scabF	scabS	sabUK	sabS	sabG	schUK	schF	schS	rubUK	rubF	rubS	rubG	rugUK	rugF	rugS	rugG	van	spe	lob	lobu	wes	sul	alo
C27:1 (all isomers)	2670-2698	0.76 ± 0.31 ***	0.75 ± 0.15 ***	0.94 ± 0.22 ***	6.44 ± 1.71	6.15 ± 1.51	12.77 ± 2.9				*t* **	0.59 ± 0.21**	0.81 ± 0.41**	1.79 ± 0.97**	0.49 ± 0.63***	0.06 ± 0.03***	0.34 ± 0.16***	0.34 ± 0.19***	1.59***	4.57 ± 1.35 **		8.89 ± 0.96 ***	*t* **	0.82 ± 0.39 *	
(*Z*)-13-C27:1	2672				100	100	100																		
4,12-diMeC26	2691																							0.32 ± 0.08	
4,8-diMeC26	2692												0.23 ± 0.12												
n-C27	2700	0.22 ± 0.14	0.23 ± 0.07	0.56 ± 0.15	0.26 ± 0.23	0.39 ± 0.16	0.34 ± 0.06	0.26 ± 0.09	0.23 ± 0.17	0.67 ± 0.2	1.92 ± 1.18	1.37 ± 0.52	2.64 ± 0.77	2.33 ± 1.04	3.24 ± 1.93	0.95 ± 0.47	3.44 ± 1.06	2.04 ± 1.5	0.54	1.89 ± 0.46	2.30 ± 0.41	1.18 ± 0.47	2.08 ± 0.55	0.68 ± 0.24	0.64 ± 0.28
unknown	2719				1.29 ± 0.45	0.96 ± 0.37	1.71 ± 0.37																		
4,8,12-triMeC26 + MeC27:1	2723										0.22 ± 0.03	0.68 ± 0.32	0.71 ± 0.12	0.65 ± 0.92											
9-;11-;13-MeC27	2733	*t*	*t*	*t*	*t*	0.34 ± 0.11	0.54 ± 0.09	0.18 ± 0.06	*t*	0.34 ± 0.17	16.72 ± 1.21	20.16 ± 5.97	21.71 ± 1.54	16.89 ± 2.18	1.58 ± 1.24	0.66 ± 0.31	0.83 ± 0.34	3.49 ± 0.78		1.84 ± 0.37	0.36 ± 0.26	1.18 ± 0.30	0.29 ± 0.11	3.22 ± 0.60	0.21 ± 0.1
7-MeC27	2742	*t*	*t*	*t*				*t*	*t*	*t*	1.83 ± 0.43	2.32 ± 1.75	1.94 ± 0.5	2.33 ± 0.23	0.41 ± 0.21	0.63 ± 0.51	0.86 ± 0.92	1.4 ± 0.71		1.01 ± 0.20		*t*	*t*		
5-MeC27	2752	*t*	*t*	*t*	1.33 ± 0.38	1.88 ± 0.52	1.76 ± 0.43	*t*	*t*	*t*	4.24 ± 1.00	5.81 ± 1.67	4.39 ± 1.05	4.41 ± 0.01	1.25 ± 0.89	0.7 ± 0.43	1.66 ± 1.21	3.61 ± 0.86		4.69 ± 0.79	*t*	0.38 ± 0.41	*t*	*t*	0.60 ± 0.08
11,15+9,13-diMeC27	2766										8.14 ± 1.04	5.86 ± 2.02	4.06 ± 1.4	4.6 ± 0.25	*t*		*t*	0.73 ± 0.24							
9,15-diMeC27	2767							*t*	*t*															0.87 ± 0.22	
7,11-diMeC27 (+3-MeC27)	2773							0.64 ± 0.31	0.69 ± 0.38	1.14 ± 0.39	4.64 ± 0.3	11.25 ± 2.18	4.66 ± 1.03	16.13 ± 2.34											
7,15-diMeC27 (+3-MeC27)	2774														0.62 ± 0.28	0.7 ± 0.59	0.71 ± 0.51	2.24 ± 0.9							
7,17-diMe (+3-MeC27)	2774																			0.94 ± 0.32					
3-MeC27	2774		*t*	*t*	*t*	*t*	*t*												0.26		3.63 ± 0.63	0.47 ± 0.21	1.83 ± 0.52	0.65 ± 0.10	*t*
5,17-diMeC27(+C28:1)	2783																			2.80 ± 0.87					
5,15-diMeC27 + 5,17-diMeC27	2784	*t*	*t*	*t*	*t*	*t*	*t*																		
5,15-diMeC27	2784																							0.34 ± 0.02	
5,11-dimeC27	2785										6.24 ± 0.57	1.44 ± 2.03	7.83 ± 2.92	1.59 ± 0.32	0.20 ± 0.19	*t*	*t*	1.20 ± 0.55							*t*
5,9, 5,15 & 5,17-diMeC27	2789											1.64 ± 1.34													
n-C28	2800									*t*					*t*	*t*	*t*	*t*		0.21 ± 0.04	*t*		*t*		
7,11,15-triMeC27	2801										0.37 ± 0.12	1.23 ± 0.88	0.23 ± 0.06	2.32 ± 0.14										0.25 ± 0.04	
7,11,17-triMeC27	2803										0.54 ± 0.06	0.71 ± 0.83	0.28 ± 0.05	1.25 ± 0.27											
3,15-diMeC27	2808											0.36 ± 0.53												0.80 ± 0.21	

Compound	RI	scabUK	scabF	scabS	sabUK	sabS	sabG	schUK	schF	schS	rubUK	rubF	rubS	rubG	rugUK	rugF	rugS	rugG	van	spe	lob	lobu	wes	sul	alo
3,9-diMeC27	2808																			*t*					
3,7-diMeC27	2810									0.55 ± 0.22												0.22 ± 0.22	0.53 ± 0.96		
5,9,13-triMeC27 (+3,7-diMeC27)	2811										2.16 ± 0.21	0.85 ± 0.43	2.24 ± 0.56	1.71 ± 0.32											
5,9,17-triMeC27	2825											0.14 ± 0.15	*t*	0.16 ± 0.01											
10-;12-;13-;14-MeC28	2834										1.4 ± 0.22	1.39 ± 0.4	1.85 ± 0.17	0.92 ± 0.03	0.56 ± 0.24	0.41 ± 0.12	0.45 ± 0.13	0.8 ± 0.15		0.35 ± 0.14					
3,7,11-triMeC27	2840										1.04 ± 0.15	0.7 ± 0.35	1.46 ± 0.4	1.05 ± 0.1											
3,7,13-triMC27 (+8-MeC28)	2840							0.71 ± 0.19	0.58 ± 0.17	1.38 ± 0.44															
8-MeC28	2840														*t*	*t*	*t*	*t*							
MeC29:2	2850	0.72 ± 0.8	*t*																*t*						
C29:2 [a]	2843-2873	0.20 ± 0.17 *	*t*	*t*											0.98 ± 0.97 [a]			0.93 ± 0.45 [a]		34.71 ± 3.90 **	0.55 ± 0.22 **		35.50 ± 5.28 *		
MeC29:2	2862																		0.62						
10,14+10,12-diMeC28	2863										0.3 ± 0.26	0.34 ± 0.11	0.20 ± 0.13	0.30 ± 0.08											
3,7,11,15-tetraMeC27	2865										0.82 ± 0.08	0.72 ± 0.59	1.14 ± 0.17	1.02 ± 0.25											
8,14-diMeC28	2869							0.95 ± 0.17	0.74 ± 0.18	1.09 ± 0.24		0.18 ± 0.26													
6,10-diMeC28	2876										0.30 ± 0.05	0.32 ± 0.09	0.26 ± 0.07	0.34 ± 0.07											
C29:1 (all isomers)	2869-2892	0.69 ± 0.51 ***	0.59 ± 0.14 ***	0.62 ± 0.19 ***	0.25 ± 0.27	0.22 ± 0.14	0.85 ± 0.32								9.15 ± 3.05	5.68 ± 1.14	10.8 ± 2.73	5.5 ± 0.9	0.71	22.83 ± 4.08 **	15.12 ± 3.82 ***	0.45 ± 0.13 *	26.15 ± 1.56**	*t* *	
(*Z*)-13-C29:1	2870														6.34	5.71	3.54	9.33							
(*Z*)-11-C29:1	2878														35.95	22.84	20.16	46.85							
(*Z*)-9-C29:1	2882														35.03	29.49	49.93	28.18							
(*Z*)-7-C29:1	2887														13.71	41.96	19.86	7.86							
(*Z*)-6-C29:1	2891														8.98	*t*	6.51	7.79							
4,12-diMeC28	2892										0.27 ± 0.09	0.16 ± 0.07	0.21 ± 0.05	0.31 ± 0.08											
4,8-diMeC28	2892							*t*	*t*	*t*															
n-C29	2900	0.19 ± 0.14	0.18 ± 0.06	0.29 ± 0.11	*t*	*t*	*t*	5.02 ± 0.48	2.79 ± 0.87	5.07 ± 2.61	0.51 ± 0.17	0.40 ± 0.17	0.76 ± 0.24	1.07 ± 0.32	1.58 ± 1.3	0.97 ± 0.21	1.96 ± 0.95	0.55 ± 0.29	0.29	1.69 ± 0.35	1.10 ± 0.77	0.59 ± 0.05	2.29 ± 0.61	*t*	*t*
MeC29:1	2920										0.02 ± 0.04	0.51 ± 0.27		0.23 ± 0	1.76 ± 1.7		0.32 ± 0.2	1.07 ± 0.37							
11-;13-MeC29	2933							1.16 ± 0.25	0.45 ± 0.3	1.4 ± 0.46											0.57 ± 0.17				

Compound	RI	scabUK	scabF	scabS	sabUK	sabS	sabG	schUK	schF	schS	rubUK	rubF	rubS	rubG	rugUK	rugF	rugS	rugG	van	spe	lob	lobu	wes	sul	alo
9-;11-;13-MeC29	2933	0.47 ± 0.53	0.16 ± 0.1	0.27 ± 0.35	*t*	*t*	0.38 ± 0.12				7.12 ± 1.37	6.8 ± 1.57	6.23 ± 1.08	7.84 ± 0.1	11.47 ± 3.58	10.98 ± 3.25	10.99 ± 2.06	15.92 ± 1.8		3.05 ± 0.59		0.45 ± 0.13	0.79 ± 0.23	0.32 ± 0.11	0.21 ± 0.09
9-MeC29	2938							0.94 ± 0.23	0.76 ± 0.18	1.15 ± 0.43											0.37 ± 0.15				
7-MeC29	2942	*t*	*t*					3.98 ± 1.15	4.72 ± 0.82	6.03 ± 1.21	0.38 ± 0.16	0.26 ± 0.14	0.74 ± 0.14	0.21 ± 0.06	*t*	0.64 ± 0.17	0.91 ± 0.29	0.53 ± 0.07		0.28 ± 0.07	0.45 ± 0.3		*t*		
4,8,12,16-tetraMeC28	2948											0.15 ± 0.19													
5-MeC29	2952	*t*	*t*		*t*	0.40 ± 0.13	0.66 ± 0.27	0.32 ± 0.07	0.27 ± 0.22	1.07 ± 0.27	0.56 ± 0.21	0.5 ± 0.2	0.92 ± 0.15	0.8 ± 0.19	1.72 ± 0.97	2.24 ± 0.7	3.58 ± 1.27	1.47 ± 0.49		1.27 ± 0.21	1.05 ± 0.08		1.04 ± 0.27		
13,17-diMeC29	2963														1.84 ± 2.33	0.34 ± 0.46	1.69 ± 0.58	7.98 ± 3.13							
13,17+11,17-diMeC29	2960										4.39 ± 0.77	5.21 ± 3.33	2.88 ± 0.53	2.5 ± 0.42											
11,15-diMeC29	2964														0.85 ± 1.9	1.31 ± 1.38									
11,17+9,17-diMeC29	-											0.57 ± 1.27													
unknown	2965																						1.47 ± 0.54		
9,15-diMeC29	2966							2.49 ± 0.13	1.48 ± 0.28	1.85 ± 0.33															
9,15+9,19-diMeC29	2968															0.60 ± 0.1	*t*								
9,13+9,17-diMeC29	-													3.46 ± 0.04											
9,17+9,19-diMeC29	-											*t*	*t*												
7,17-diMeC29 (+3-MeC29)	2972																			0.53 ± 0.16				*t*	
7,11-diMeC29 (+3-MeC29)	2973							5.35 ± 1.84	5.72 ± 1.02	7.05 ± 0.89	1.49 ± 0.36	2.15 ± 1.03	1.11 ± 0.31	2.64 ± 0.11											
7,19-diMeC29 (+3-MeC29)	2976														2.02 ± 0.28	1.88 ± 0.63	2.74 ± 0.67	2.72 ± 0.22							
3-MeC29	2975		*t*	*t*																	1.40 ± 0.41		2.32 ± 0.85		
5,9-diMeC29	2983							0.67 ± 0.18	0.49 ± 0.41	0.9 ± 0.28															
5,17-diMeC29	2983	0.47 ± 0.6	*t*	0.19 ± 0.15	*t*	*t*	*t*										1.62 ± 1.2			2.25 ± 0.76	0.20 ± 0.14		0.73 ± 0.20		
5,11+5,17-diMeC29	2983										2.65 ± 0.45	1.60 ± 0.44	2.43 ± 0.5	0.85 ± 0.03											
5,15+5,17-diMeC29	2985														1.64 ± 0.99	1.23 ± 1.16		6.48 ± 2.09							
9,13,17-triMeC29	-											0.20 ± 0.29		0.66 ± 0.01											
7,11,15-triMeC29	2995											0.35 ± 0.28													
7,11,17-triMeC29	3000										0.33 ± 0.05	0.71 ± 0.85		2.03 ± 0.12											

Compound	RI	scabUK	scabF	scabS	sabUK	sabS	sabG	schUK	schF	schS	rubUK	rubF	rubS	rubG	rugUK	rugF	rugS	rugG	van	spe	lob	lobu	wes	sul	alo
n-C30	3000							1.46 ± 0.12	0.9 ± 0.07	1.14 ± 0.54															
5,9,11-triMeC29	-											0.11 ± 0.09		0.20 ± 0											
5,9,13 or 5,9,15-triMeC29	3007												0.49 ± 0.08												
3,15-diMeC29	3008										0.74 ± 0.18	*t*			0.74 ± 0.29	0.61 ± 0.23	1.16 ± 1.09	1.08 ± 0.41							
3,7-diMeC29	3009							1.55 ± 0.23	2.17 ± 0.23	3.7 ± 0.68										*t*			0.34 ± 0.43		
12-;13-;14-MeC30	3032							0.71 ± 0.04	0.38 ± 0.08	0.74 ± 0.17	0.45 ± 0.03	0.21 ± 0.06	0.20 ± 0.03	0.16 ± 0.02	0.75 ± 0.15	1.05 ± 0.2	1.22 ± 0.3	0.77 ± 0.06							
3,7,11-triMeC29	3039										0.31 ± 0.04	0.27 ± 0.19	0.53 ± 0.44	0.36 ± 0.02											
8-MeC30	3040							3.31 ± 0.34	4.13 ± 0.39	4.72 ± 0.4															
MeC31:2	3048	0.85 ± 0.74	*t*																						
MeC31:2	3056																		0.94						
13,17-diMeC30	3060									0.32 ± 0.62		0.18 ± 0.09		*t*											
3,7,11,17-tetraMeC29	3062										0.49 ± 0.05	0.17 ± 0.14	0.33 ± 0.14	0.30 ± 0.01											
8,14-diMeC30	3068							2.8 ± 0.16	2.78 ± 0.3	2.37 ± 0.58															
C31:2 [a]	3047-3080														21.23 ± 4.63	1.71 ± 0.64	18.59 ± 3.93	6.73 ± 1.84		0.85 ± 0.19 ***	60.11 ± 1.57 **		18.12 ± 3.54 **		*t* *
6,10-diMeC30	3075							0.54 ± 0.09	0.56 ± 0.08	0.74 ± 0.09															
C31:1 (all isomers)	3070-3090	0.43 ± 0.32	0.30 ± 0.17	0.23 ± 0.13	*t*	*t*	*t*								1.71 ± 1.79	8.32 ± 2.35	2.9 ± 1.73	3.02 ± 1.13	0.48 *	0.23 ± 0.09*	12.04 ± 2.49 *		4.90 ± 0.85 **		
(*Z*)-13-C31:1	3070														12.18	8.95	5.25	13.53							
(*Z*)-11-C31:1	3077														55.65	54.58	51.21	63.94							
(*Z*)-9-C31:1	3082														32.17	21.95	38.87	22.53							
(*Z*)-7-C31:1	3088														*t*	14.52	4.67	*t*							
4,8-diMeC30	3091							*t*	*t*	*t*															
n-C31	3100	*t*	*t*	*t*				4.44 ± 1.28	1.9 ± 0.46	2.9 ± 1.4									*t*						*t*
MeC31:1	-										*t*	0.21 ± 0.17		*t*	0.53 ± 0.52		0.54 ± 0.34	0.58 ± 0.28							
MeC31:1	-														2.71 ± 1.3		2.13 ± 0.8	1.1 ± 0.49							
11-;13-;15-MeC31	3134							7.49 ± 0.99	5.21 ± 0.57	6.44 ± 1.49	1.84 ± 0.9	0.64 ± 0.17	0.52 ± 0.15	0.59 ± 0.09	5.81 ± 1.27	9.96 ± 2.63	5.99 ± 1.46	3.61 ± 0.95		0.42 ± 0.35	0.61 ± 0.12	0.24 ± 0.15	0.27 ± 0.05		*t*
9-MeC31	3139							3.84 ± 0.51	4.37 ± 0.57	3.44 ± 0.65															

Compound	RI	scabUK	scabF	scabS	sabUK	sabS	sabG	schUK	schF	schS	rubUK	rubF	rubS	rubG	rugUK	rugF	rugS	rugG	van	spe	lob	lobu	wes	sul	alo
7-MeC31	3145							3.79 ± 0.62	4.23 ± 0.57	4.5 ± 0.77															
4,8,12,16-tetraMeC30	-										0.22 ± 0.08	*t*	0.16 ± 0.07												
5-MeC31	-														0.88 ± 0.25	0.64 ± 0.27	1.02 ± 1.0								
13,17-diMeC31	3161							1.12 ± 0.2	0.81 ± 0.12	2.65 ± 1.8	2.52 ± 0.11	1.68 ± 2.06	0.93 ± 0.32	0.91 ± 0.04	5.9 ± 3.52	9.01 ± 3.99	9.88 ± 3.64	8.67 ± 0.91		*t*					
11,19-diMeC31	-											0.31 ± 0.22	*t*	0.39 ± 0.02									0.28 ± 0.17		
11,15-diMeC31	-																								
9,15-diMeC31	3169							4.89 ± 0.55	3.01 ± 0.64	2.65 ± 0.45															
9,x-diMeC31 (mixture of 9,13; 9,17; 9,19; 9,21)	-														1.89 ± 0.86	1.74 ± 0.51	1.45 ± 0.7	1.14 ± 0.2							
9,x-diMeC31+3-MeC31	-											*t*	*t*	0.30 ± 0.06											
MeC33:2	3175	*t*																							
7,19-diMeC31 +3-MeC31	-										0.21 ± 0.03	*t*	0.27 ± 0.07												
7,11+7,17+7,19-diMeC31+(3-MeC31)	3178							16.0 ± 1.17	21.85 ± 2.1	15.3 ± 1.21															
7,x-diMeC31 +3-MeC31	-															1.17 ± 0.51	0.44 ± 0.58	0.6 ± 0.13							
5,17-diMeC31	3184	*t*			*t*	*t*	*t*	1.52 ± 0.36	0.96 ± 0.33	1.35 ± 0.76	4.08 ± 2.62	0.16 ± 0.08	0.20 ± 0.12	*t*	2.38 ± 1.43	2.45 ± 2.45	1.70 ± 1.71	5.02 ± 0.51							
5,15-diMeC31	-																			0.35 ± 0.42					
9,13,17-triMeC31	-											*t*	*t*	*t*											
7,11,13 or 7,11,17-triMeC31	3201							1.92 ± 0.16	2.32 ± 0.27	1.75 ± 0.24															
7,11,x-triMeC31	-										1.47 ± 1.27	0.03 ± 0.05													
3,15-diMeC31	-											*t*		0.39 ± 0.01	0.77 ± 0.33	0.95 ± 0.24	0.78 ± 0.82	0.79 ± 0.07							
3,7-diMeC31	3214							0.85 ± 0.23	1.15 ± 0.25	1.36 ± 0.22															
unknown	3218							1.1 ± 0.2	1.63 ± 0.34	0.75 ± 0.1															
14-;15-;16-MeC32	3233							1.13 ± 0.11	1.08 ± 0.08	0.95 ± 0.12															
14-MeC32	-														*t*	0.28 ± 0.27	*t*								
12-;14-MeC32	-										0.34 ± 0.23														
3,7,15-triMeC31	-										0.48 ± 0.21														
3,7,13-triMeC31 (+8-MeC32)	3242							2.14 ± 0.14	3.29 ± 0.46	2.65 ± 0.31															

Compound	RI	scabUK	scabF	scabS	sabUK	sabS	sabG	schUK	schF	schS	rubUK	rubF	rubS	rubG	rugUK	rugF	rugS	rugG	van	spe	lob	lobu	wes	sul	alo
C33:2	-														5.7 ± 0.36	11.87 ± 4.63	4.31 ± 1.51	2.33 ± 0.89			1.58 ± 0.46 ***				
4,18-diMeC32	3262							*t*	0.21 ± 0.1	0.28 ± 0.31															
8,14-diMeC32	3270							3.63 ± 0.3	4.51 ± 0.6	2.96 ± 0.5															
6,14-diMeC32	-										0.21 ± 0.14														
C33:1 [a]	-														0.88 ± 0.42	1.37 ± 0.15	0.85 ± 0.22	0.29 ± 0.12			*t*				
4,14+4,16-diMeC32	-										0.40 ± 0.45														
C33	3300							0.38 ± 0.12	0.22 ± 0.06																
MeC33:1	-														0.57 ± 0.27	0.60 ± 0.65	0.28 ± 0.13	0.39 ± 0.29							
MeC33:1	-														0.30 ± 0.2	0.19 ± 0.2	0.20 ± 0.09	*t*							
13-;15-;17-MeC33	-							2.31 ± 0.47	2.23 ± 0.26	1.8 ± 0.21															
9-;11-;13-MeC33	-														0.68 ± 0.23	1.26 ± 0.39	0.53 ± 0.29	0.66 ± 0.18							
11-MeC33	-										0.86 ± 0.94														
9-MeC33	-							1.19 ± 0.18	1.33 ± 0.15	1.07 ± 0.26															
13,17-diMeC33	-							0.56 ± 0.09	0.40 ± 0.05	0.80 ± 0.6					0.68 ± 0.22	1.05 ± 0.2	1.08 ± 1.1	0.79 ± 0.11							
11,21-diMeC33	-														0.69 ± 0.27	1.05 ± 0.2	0.52 ± 0.67	0.55 ± 0.15							
9,19+9,21-diMeC33	-							4.13 ± 0.44	3.83 ± 0.25	2.07 ± 0.2															
7,19-diMeC33	-							1.7 ± 0.23	2.86 ± 0.45	1.45 ± 0.36															
5,15-diMeC33	-																			*t*					
5,17-diMeC33	-														0.69 ± 0.46	1.05 ± 0.59	0.44 ± 0.48	1.15 ± 0.28							
5,19-diMeC33	-										1.98 ± 2.39														
7,13,19-triMeC33	-							0.52 ± 0.02	0.67 ± 0.16	*t*															
unknown	-							0.37 ± 0.14	0.58 ± 0.22																
14-;15-;16-MeC34	-							0.20 ± 0.57	*t*																
3,7,13-triMeC33	-							*t*	*t*																
3,x,y-triMe +3,15-diMeC33	-										0.37 ± 0.45														
8,14-diMeC34	-							0.53 ± 0.13	0.54 ± 0.12																

Compound	RI	scabUK	scabF	scabS	sabUK	sabS	sabG	schUK	schF	schS	rubUK	rubF	rubS	rubG	rugUK	rugF	rugS	rugG	van	spe	lob	lobu	wes	sul	alo
C35:2 [a]	-														1.2 ± 0.31	6.97 ± 1.87	0.35 ± 0.27	0.5 ± 0.25			*t*				
C35:1	-															0.90 ± 0.2		*t*							
13-;15-;17-MeC35	-							0.38 ± 0.03	0.40 ± 0.09																
11-;13-;15-MeC35	-															0.34 ± 0.07		*t*							
13,17+11,x-diMeC35	-														0.33 ± 0.19	1.03 ± 0.15	0.19 ± 0.16	0.61 ± 0.08							
9,21-diMeC35	-							1.22 ± 0.33	0.85 ± 0.11																
unknown	-							0.55 ± 0.18	0.43 ± 0.18																
5,17-diMeC35	-														*t*	0.44 ± 0.15		0.24 ± 0.06							
C37:2 [a]	-														0.28 ± 0.17	2.62 ± 0.82									
C37:1	-															0.33 ± 0.20									
13,17+11,x-diMeC37	-														0.20 ± 0.16	0.52 ± 0.12		0.22 ± 0.16							
C39:2 [a]	-														0.47 ± 0.33	0.41 ± 0.38									

van Den Dool H, Kratz PD (1963) A generalization of the retention index system including linear temperature programmed gas-liquid partition chromatography. J Chromatogr A 11:463-471
