# Supplementary material for: Species-Specific Cuticular Hydrocarbon Stability within European Myrmica Ants
Source: J Chem Ecol. 2016 Nov 10;42(10):1052–62. doi: 10.1007/s10886-016-0784-x (PMC5119849; doi:10.1007/s10886-016-0784-x)
Supplement: Supplementary file 3 — (DOCX 19 kb) [file 10886_2016_784_MOESM3_ESM.docx]

SPECIES-SPECIFIC CUTICULAR HYDROCARBON STABILITY WITHIN EUROPEAN *Myrmica*

ANTS

RHIAN M. GUILLEM ^1, 2,^ *, FALKO P. DRIJFHOUT ^3^, STEPHEN J. MARTIN ^4^

*^1^Department of Animal and Plant Sciences, University of Sheffield, Sheffield S10 2TN, UK*

*^2^Department of Life & Earth Sciences, Gibraltar Botanic Gardens Campus, University of Gibraltar,*

*Gibraltar*

*^3^Chemical Ecology Group, School of Physical and Geographical Sciences, Lennard-Jones Laboratory,*

*Keele University, Keele ST5 5BG, UK*

*^4^School of Environment & Life Sciences, University of Salford, Manchester M5 4WT, UK*

* Corresponding author. E-mail: rguillem@gibraltargardens.gi

**Online Resource 3** Average % similarities ±SD calculated from the bray-curtis dissimilarity distances performed on the transformed proportional data based on 22791 pairwise comparisons.

|  |  | scabUK | | scabF | | scabS | | sabUK | | sabG | | sabS | | | schS | schF | schUK | rugF | rugG | rugS | rugUK | rubF | rubUK | rubG | rubS |
| --- | --- | --- | --- | --- | --- | --- | --- | --- | --- | --- | --- | --- | --- | --- | --- | --- | --- | --- | --- | --- | --- | --- | --- | --- | --- |
|  | scabUK | 81.0 | ± 0.09 |  |  |  |  |  |  |  |  |  |  |  |  |  |  |  |  |  |  |  |  |  |  |
|  | scabF | 74.5 | ± 0.08 | 84.2 | ± 0.08 |  |  |  |  |  |  |  |  |  |  |  |  |  |  |  |  |  |  |  |  |
|  | scabS | 71.4 | ± 0.10 | 79.0 | ± 0.08 | 80.2 | ± 0.10 |  |  |  |  |  |  |  |  |  |  |  |  |  |  |  |  |  |  |
|  | sabUK | 55.0 | ± 0.06 | 51.1 | ± 0.07 | 52.4 | ± 0.10 | 90.5 | ± 0.04 |  |  |  |  |  |  |  |  |  |  |  |  |  |  |  |  |
|  | sabG | 51.1 | ± 0.05 | 49.2 | ± 0.05 | 50.4 | ± 0.09 | 87.2 | ± 0.03 | 93.3 | ± 0.02 |  |  |  |  |  |  |  |  |  |  |  |  |  |  |
|  | sabS | 53.0 | ± 0.05 | 52.6 | ± 0.06 | 55.4 | ± 0.09 | 85.6 | ± 0.02 | 87.0 | ± 0.02 | 92.5 | ± 0.02 |  |  |  |  |  |  |  |  |  |  |  |  |
|  | schS | 2.2 | ± 0.01 | 2.0 | ± 0.01 | 2.6 | ± 0.01 | 2.1 | ± 0.01 | 4.2 ± 0 | | 3.7 | ± 0.01 |  | 92.3 ± 0.02 |  |  |  |  |  |  |  |  |  |  |
|  | schF | 1.8 | ± 0.03 | 1.9 ± 0 | | 2.0 ± 0 | | 1.6 | ± 0.01 | 2.7 | ± 0.01 | 2.5 | ± 0.01 |  | 84.8 ± 0.02 | 94.6 ± 0.02 |  |  |  |  |  |  |  |  |  |
|  | schUK | 2.1 | ± 0.01 | 1.9 ± 0 | | 2.1 ± 0 | | 1.8 | ± 0.01 | 3.1 ± 0 | | 3.0 | ± 0.01 |  | 86.4 ± 0.02 | 90.8 ± 0.01 | 95.0 ± 0.02 |  |  |  |  |  |  |  |  |
|  | rugF | 8.4 | ± 0.03 | 7.7 | ± 0.01 | 8.2 | ± 0.02 | 6.6 | ± 0.02 | 10.1 | ± 0.01 | 8.5 | ± 0.02 |  | 26.0 ± 0.03 | 21.0 ± 0.02 | 23.8 ± 0.01 | 87.1 ± 0.03 |  |  |  |  |  |  |  |
|  | rugG | 10.7 | ± 0.03 | 9.7 | ± 0.01 | 9.8 | ± 0.02 | 8.7 | ± 0.02 | 12.6 | ± 0.01 | 11.1 | ± 0.02 |  | 22.6 ± 0.03 | 18.0 ± 0.01 | 20.2 ± 0.01 | 69.4 ± 0.03 | 90.4 ± 0.02 |  |  |  |  |  |  |
|  | rugS | 10.5 | ± 0.03 | 9.7 | ± 0.01 | 10.2 | ± 0.02 | 9.0 | ± 0.02 | 12.9 | ± 0.01 | 11.2 | ± 0.02 |  | 25.1 ± 0.03 | 20.0 ± 0.02 | 22.5 ± 0.02 | 68.2 ± 0.04 | 74.6 ± 0.04 | 84.3 ± 0.05 |  |  |  |  |  |
|  | rugUK | 10.1 | ± 0.03 | 9.2 | ± 0.01 | 9.5 | ± 0.02 | 8.2 | ± 0.02 | 11.9 | ± 0.02 | 10.3 | ± 0.02 |  | 22.6 ± 0.03 | 18.0 ± 0.02 | 20.5 ± 0.02 | 70.1 ± 0.03 | 75.4 ± 0.03 | 78.3 ± 0.05 | 81.2 ± 0.04 |  |  |  |  |
|  | rubF | 10.8 | ± 0.02 | 10.9 | ± 0.01 | 12.3 | ± 0.02 | 21.5 | ± 0.02 | 23.5 | ± 0.02 | 23.4 | ± 0.02 |  | 16.2 ± 0.02 | 13.2 ± 0.02 | 14.2 ± 0.02 | 21.1 ± 0.03 | 29.3 ± 0.03 | 25.9 ± 0.03 | 24.6 ± 0.03 | 80.1 ± 0.06 |  |  |  |
|  | rubUK | 8.7 | ± 0.02 | 8.6 | ± 0.01 | 10.1 ± 0.02 | | 18.0 | ± 0.02 | 19.8 | ± 0.02 | 19.9 | ± 0.02 |  | 19.6 ± 0.02 | 16.0 ± 0.02 | 17.7 ± 0.01 | 27.1 ± 0.02 | 34.1 ± 0.02 | 29.4 ± 0.02 | 28.7 ± 0.03 | 70.9 ± 0.06 | 88.0 ± 0.03 |  |  |
|  | rubG | 11.0 | ± 0.01 | 11.2 | ± 0.01 | 12.8 | ± 0.02 | 21.4 | ± 0.01 | 23.2 | ± 0.01 | 23.3 | ± 0.02 |  | 16.6 ± 0.01 | 13.8 ± 0.01 | 14.8 ± 0.01 | 22.5 ± 0.02 | 30.8 ± 0.02 | 27.2 ± 0.01 | 26.2 ± 0.02 | 78.3 ± 0.03 | 70.3 ± 0.03 | 94.0 ± 0.06 |  |
|  | rubS | 12.1 | ± 0.02 | 12.4 | ± 0.01 | 14.2 | ± 0.02 | 22.7 | ± 0.02 | 25.2 | ± 0.02 | 25.2 | ± 0.03 |  | 16.5 ± 0.01 | 13.6 ± 0.02 | 14.5 ± 0.01 | 22.0 ± 0.02 | 29.0 ± 0.02 | 25.8 ± 0.02 | 24.0 ± 0.02 | 72.7 ± 0.04 | 79.1 ± 0.04 | 73.1 ± 0.02 | 90.4 ± 0.03 |
